# Investigation of the Effects of Postbiotics Obtained from *Pediococcus acidilactici* on Specific Biomarker Expressions in Intestinal Tissue

**DOI:** 10.3390/foods15071267

**Published:** 2026-04-07

**Authors:** Ismail Demircioğlu, Muhammet Bahaeddin Dörtbudak, Funda Aksünger Karaavci, Mehmet Emin Aydemir, Muhammed Demircioğlu, Aydın Genç, Ayşegül Demircioğlu, Güven Güngör, Alessandro Di Cerbo

**Affiliations:** 1 Department of Anatomy, Faculty of Veterinary Medicine, Harran University, 63000 Şanlıurfa, Türkiye; 2 Department of Pathology, Faculty of Veterinary Medicine, Harran University, 63000 Şanlıurfa, Türkiye; 3 Department of Anatomy, Faculty of Veterinary Medicine, Bingöl University, 12000 Bingöl, Türkiye; 4 Department of Food Hygiene and Technology, Faculty of Veterinary Medicine, Harran University, 12000 Şanlıurfa, Türkiye; 5 Department of Histology and Embryology, Faculty of Medicine, İstanbul Aydın University, 34000 Istanbul, Türkiye; 6 Department of Biochemstry, Faculty of Veterinary Medicine, Bingöl University, 12000 Bingöl, Türkiye; 7 Department of Food Hygiene and Technology, Faculty of Veterinary Medicine, Bursa Uludağ University, 16000 Bursa, Türkiye; 8 Department of Biostatistics, Faculty of Veterinary Medicine, Bingöl University, 12000 Bingöl, Türkiye; ggungor@bingol.edu.tr; 9 School of Bioscience and Veterinary Medicine, University of Camerino, 62024 Matelica, Italy

**Keywords:** intestine, biomarker, immunohistochemistry, postbiotic

## Abstract

The intestinal mucosal barrier is a layered structure comprising fundamental components that play important roles in regulating paracellular permeability. Disruption of intestinal barrier homeostasis predisposes to infections, mucosal damage, and metabolic and allergic diseases. To provide protection against potential damage to the intestinal mucosa, agents such as prebiotics and probiotics are recommended due to their ability to secrete components and metabolites (e.g., bacteriocins, organic acids, enzymes) that can exert beneficial biological effects. The aim of this study is to comprehensively investigate the effects of a postbiotic derived from *Pediococcus acidilactici* on healthy rat intestinal tissue. A total of 78 Wistar Albino rats were used in this study. Following compositional analysis of the postbiotic, the animals were administered the postbiotic orally via gavage for different durations (7, 14, 21, 28 days) and at different doses (250 mg/Kg, 500 mg/Kg, 1000 mg/Kg). Characterization of the produced postbiotic revealed a diverse spectrum of biologically active compounds, including organic acids, phenolics, and volatile compounds. Histopathological examination of intestinal sections (duodenum, jejunum, ileum, cecum, colon, and rectum) showed no pathological lesions in any of the experimental groups. Conversely, immunohistochemical analysis revealed that the postbiotic increased the expression of CLDN3, OCLN, ZO1, AQP4, and AQP8, proteins involved in intestinal permeability and fluid transport, in a dose-dependent manner. These results highlight the potential of *Pediococcus acidilactici* as a supportive agent in a range of intestinal pathologies, including major intestinal diseases such as Crohn’s disease, ulcerative colitis, and inflammatory bowel disease (IBD).

## 1. Introduction

The intestinal mucosal barrier is a layered structure comprising fundamental components such as the mucus and epithelial layers; the underlying lamina propria; and layers containing tight junction proteins, immune cells, and antimicrobial peptides. These components play important roles in regulating paracellular permeability [1,2]. Disruption of intestinal barrier integrity predisposes individuals to infections, mucosal damage, and metabolic and allergic diseases [1]. Maintaining intestinal homeostasis and mucosal barrier integrity is important not only for gastrointestinal health but also for neurological function. Disruption of the intestinal flora can impair the regulation of corticotropin and cortisol production, thereby affecting neuroendocrine function [3].

Transepithelial fluid transport in the intestines occurs via paracellular and transcellular pathways. In paracellular transport, tight junctions in the intestinal epithelium regulate passage based on the size and charge of transported substances. In transcellular transport, fluid movement occurs via aquaporins [4]. Tight junctions are protein complexes localized at the apical region of the lateral wall of intestinal epithelial cells and are responsible for regulating the paracellular passage of ions, solutes, and fluids [5]. This protein complex comprises occludin (OCLN), claudin (CLDN), junctional adhesion molecules (JAMs), and tricellulin. These proteins form a selective barrier through homophilic and heterophilic interactions with adjacent cells [5,6]. Aquaporins, which mediate transcellular transport, are specialized membrane proteins involved in the transport of water and small solutes.

They represent the primary cellular pathway for bidirectional fluid transport in the intestines and play an important role in fluid reabsorption in the colon [7,8,9]. Aquaporin 4 (AQP4) and aquaporin 8 (AQP8) have been immunolocalized in all intestinal segments (duodenum, jejunum, ileum, cecum, colon, and rectum) in humans, rats, pigs, and rabbits [10,11]. Mucosal barrier dysfunction is a key contributor to many gastrointestinal diseases, particularly Crohn’s disease [12]. For this reason, maintaining intestinal hemostasis and preserving mucosal integrity are of great importance. For this purpose, agents such as prebiotics and probiotics are recommended [13,14]. The presence of SlpA, a surface-layer protein of probiotics, contributes to the protection of the healthy gastrointestinal microbiota and the intestinal mucosal barrier [15].

While prebiotics nourish live probiotics and help them survive, postbiotics, the biochemical byproducts produced at the end of this digestive process, provide the primary functional benefits for overall health. Postbiotics are defined as the secreted components and metabolites of probiotics that exert biological effects and are associated with symbiotic interactions between probiotics and their hosts [16]. Their composition includes macromolecules and micromolecules, such as inactivated microbial cells, cell fractions, metabolites, organic acids, bacteriocins, and enzymes. *Pediococcus* spp., members of the *Lactobacillaceae* family, are currently used as a probiotic in sausage, yogurt, and cheese production. It has been reported that, owing to the bacteriocins they produce, these bacteria inhibit the growth of pathogenic microorganisms in the host and act as signaling peptides that regulate host health [17]. Compared with commonly used probiotic genera such as *Lactobacillus*, *Pediococcus* (*P.*) *acidilactici* has attracted increasing attention due to its strong capacity for bacteriocin production, particularly pediocin-like antimicrobial peptides. These bacteriocins exhibit potent inhibitory activity against several pathogenic microorganisms. Moreover, *P. acidilactici* strains are known for their high technological stability, tolerance to acidic environments, and ability to maintain metabolic activity under industrial fermentation conditions. For these reasons, this species has been widely used in fermented food production and is considered a promising source of bioactive metabolites and postbiotic compounds with potential health-promoting effects.

In light of this information, the present study aimed to investigate the potential effects of a *P. acidilactici* postbiotic on intestinal homeostasis by examining the expression of key proteins.

## 2. Materials and Methods

### 2.1. Production of Postbiotics from Pediococcus acidilactici Bacteria

Postbiotic production was carried out according to the method described by İncili et al. (2023) [18]. *P. acidilactici* (Lactoferm B-LC-78) cultures were purchased from Christian Hansen Laboratories (Copenhagen, Denmark). The culture was grown in De Man, Rogosa, and Sharpe (MRS) broth (Merck, Darmstadt, Germany) under anaerobic conditions at 37 °C for 48 h, reaching a density of 9.0 log CFU/mL. Additionally, inoculations were performed on MRS agar plates to assess bacterial viability and colony counts. After incubation, the supernatant was centrifuged at 4200× *g* for 10 min at 4 °C. The pellet containing bacterial cells was discarded, and the supernatant was collected and passed through a 0.45 μm membrane filter (MF-Millipore, Merck, Darmstadt, Germany) to obtain a cell-free fraction. The supernatant was filtered through 0.45 μm filters. The resulting filtrate, which was free of bacterial cells but contained microbial metabolites, was then lyophilized at −80 °C for 24 h in a vacuum lyophilizer (Teknosem TRS-2-2, Istanbul, Turkey) to obtain a postbiotic preparation. After lyophilization, the total yield was calculated. The freshly obtained postbiotics were diluted with sterile distilled water to the desired concentrations for the study (250 mg/Kg, 500 mg/Kg, 1000 mg/Kg).

### 2.2. Postbiotics Characterization

#### 2.2.1. Total Phenolic Content (TPC) Determination

TPC was determined following the method of Aydemir et al. (2025) [19]. Gallic acid (ACS reagent, ≥98.0%, Merck, Darmstadt, Germany) was used as the standard. The concentration of total phenolic compounds in the samples was expressed as mg gallic acid equivalents (GAE)/mL.

#### 2.2.2. Antioxidant Activity (AA) Determination

The antioxidant capacity of the prepared postbiotics was determined by measuring their 2,2-diphenyl-1-picrylhydrazyl (DPPH) and 2,2-azino-bis(3-ethylbenzothiazoline-6-sulfonic acid) (ABTS) radical-scavenging activities according to the method proposed by Aydemir et al. (2024) [20]. The results were expressed as IC_50_.

#### 2.2.3. Organic Acids Determination

The determination of organic acids in postbiotic samples was performed using a modified version of the method reported by Aydemir et al. (2025) [21]. A reverse-phase high-performance liquid chromatography (RP-HPLC) system (Shimadzu LC-20AD, Kyoto, Japan) equipped with a diode array detector (SPD-M20A) was used for the analyses. An organic acid Mix12 solution was used as the standard mixture, and quantification was performed by comparing the peak areas of the identified organic acids with the corresponding calibration curves. The results were expressed as mg/mL per sample.

#### 2.2.4. Phenolic Compounds Analysis

The determination of phenolic compounds was performed with slight modifications to the method reported by Aydemir et al. (2025) [21]. An HPLC system (Shimadzu LC-20AD, Kyoto, Japan) equipped with a diode array detector was used for the analyses. Identification was carried out by comparing retention times and UV spectra with those of standard phenolic compounds (e.g., chlorogenic acid, caffeic acid, vanillin, *p*-coumaric acid, naringin, and resveratrol). Quantitative results were calculated as mg/mL in the sample.

#### 2.2.5. Volatile Compounds Identification

The profile of volatile compounds in postbiotic samples was determined using gas chromatography-mass spectrometry (GC-MS) (Shimadzu QP2010, Kyoto, Japan). Analyses were performed according to the method reported by İncili et al. (2025) [22]. Volatile compounds were separated using a capillary column (HP-5MS 60 m × 0.25 mm × 0.25 μm, Agilent J&W GC columns, Santa Clara, CA, USA). Helium was used as the carrier gas. The oven temperature was initially set at 50 °C, increased to 300 °C at a rate of 3 °C/min, and then held at 300 °C for 10 min. During the analytical run, the ion source was set to 200 °C, the EM voltage to 0.92 kV, and the solvent cut time to 2 min. Scan mode was used, and acquisition was performed from 2.4 to 93.0 min over a mass range of 40–700 m/z. Volatile compounds were identified by comparing their mass spectra with the NIST library and evaluating their retention times. The results were expressed as peak area percentages (%).

### 2.3. Animals and Experimental Setup

The study used male Wistar albino rats (*n* = 78) weighing approximately 250 g and aged 6–10 weeks. The animals were maintained at 22 °C under a 12:12 h light-dark cycle and were provided with food and water *ad libitum*. The animals were randomly assigned to 13 groups, with six rats per group, as follows:

Group 1 (control): MRS broth was administered via gavage for 28 days.
Group 2: 250 mg/kg postbiotic daily for 7 days.
Group 3: 250 mg/kg postbiotic daily for 14 days.
Group 4: 250 mg/kg postbiotic daily for 21 days.
Group 5: 250 mg/kg postbiotic daily for 28 days.
Group 6: 500 mg/kg postbiotic daily for 7 days.
Group 7: 500 mg/kg postbiotic daily for 14 days.
Group 8: 500 mg/kg postbiotic daily for 21 days.
Group 9: 500 mg/kg postbiotic daily for 28 days.
Group 10: 1000 mg/kg postbiotic daily for 7 days.
Group 11: 1000 mg/kg postbiotic daily for 14 days.
Group 12: 1000 mg/kg postbiotic daily for 21 days.
Group 13: 1000 mg/kg postbiotic daily for 28 days.

This study was conducted at the Bingöl University Animal Experiment Application and Research Center Laboratory Animals Unit with the approval of the Bingöl University Animal Experiments Local Ethics Committee (Meeting Number: 2024/04; Decision Number: 04/01). At the end of the treatment period of each group’s treatment period, all animals were sacrificed under anesthesia via intraperitoneal injection of thiopental sodium (40 mg/kg; Pental Sodium, I.E. Ulugay, Turkey), followed by necropsy.

### 2.4. Histomorphological Examination

Following necropsy, tissue samples obtained from different intestinal segments, including the duodenum, jejunum, ileum, cecum, colon, and rectum, were fixed in 10% buffered formalin solution. After fixation, the tissues were washed under running tap water and subjected to the routine histopathological processing procedure (dehydration, clearing, and paraffin infiltration). Subsequently, the processed tissues were embedded in paraffin blocks, and 4 µm thick sections were cut using a rotary microtome. The sections were then mounted on standard slides for histopathological examination and adhesive-coated slides for immunohistochemical analysis. Tissue sections mounted on normal slides were stained with hematoxylin-eosin (H&E) for histological examination. For this purpose, the sections were incubated in an oven for approximately 1 h and then passed through a xylene series for deparaffinization. The tissues were subsequently rehydrated through graded ethyl alcohol solutions of decreasing concentration and rinsed in distilled water before being stained with hematoxylin. After hematoxylin staining, the sections were rinsed under running tap water and then counterstained with eosin. The eosin-stained tissues were rapidly passed through increasing concentrations of alcohol, then immersed in absolute ethyl alcohol for a short period. Finally, the sections were cleared in a xylene series, mounted with Entellan, and covered with a coverslip. The hematoxylin-eosin-stained sections were examined under a light microscope [23].

### 2.5. Immunohistochemical Examination

Tissue sections mounted on adhesive slides for immunohistochemical staining were incubated in an oven for approximately 1 h; then sequentially deparaffinized; and rehydrated by passing through xylene, graded alcohol series, and distilled water. The tissues were then washed in distilled water and incubated in 3% H_2_O_2_ to inactivate endogenous peroxidase. After washing with phosphate-buffered saline (PBS; 0.01 M, pH 7.4), antigen retrieval was performed by boiling the sections in citrate buffer (0.01 M, pH 6.0) and allowing them to cool; this process was repeated three times. The tissues were then washed again with PBS, encircled with a PAP pen, and incubated in a humidified chamber after the application of a protein block solution to prevent non-specific binding. Without further washing, primary antibodies targeting the biomarkers evaluated in the study: CLDN3 (Thermo Fisher, Waltham, MA, USA; 34-1700; 1/50), OCLN (Thermo Fisher Scientific, Waltham, MA, USA; 71-1500; 1/50), ZO-1 (Thermo Fisher, Waltham, MA, USA; Cat. 61-7300; 1/50), AQP4 (Thermo Fisher Scientific, Waltham, MA, USA; Cat. No. PA5-53234; 1/50), and AQP8 (Thermo Fisher Scientific, Waltham, MA, USA; PA5-103616; 1/50), were applied to the sections and incubated overnight at 4 °C. For negative control staining, primary antibodies were omitted from a subset of tissue sections. Following incubations, the sections were washed with PBS and incubated with a biotinylated secondary antibody. After another PBS wash, the sections were treated with a streptavidin-peroxidase conjugate. The tissues were then washed again with PBS and incubated with 3-3′ Diaminobenzidine (DAB) chromogen to visualize the immunoreaction and obtain the desired staining intensity. The immunostained sections were counterstained with Mayer’s hematoxylin. After dehydration through an alcohol-xylol series, a drop of Entellan was applied, and the slides were coverslipped. Immunohistochemically stained sections were examined under a light microscope, and protein expression intensity in intestinal tissues was evaluated using a five-point scoring system (1 = very weak, 2 = weak, 3 = moderate, 4 = strong, 5 = very strong) [24,25].

### 2.6. Statistical Analysis

Data were analyzed using GraphPad Prism 9 software (GraphPad Software, Inc., La Jolla, CA, USA). In this study, a five-point scoring system (1–5) was used to evaluate immunohistochemical results [25]. Scoring was performed by two independent experts to minimize observer bias. Agreement between the evaluators was assessed using the Kappa statistic. The calculated Kappa values (CLDN3: 0.72, OCLN: 0.67, ZO1: 0.74, AQP4: 0.66, AQP8: 0.71) were statistically significant and fell within the ‘strong’ (substantial) agreement category according to the classification of Landis and Koch’s (1977) [26]. These results support the reliability of the scoring methodology.

Ordinal logistic regression analysis was performed to examine the combined effect of dose and time on the immunohistochemically measured expression levels of CLDN3, OCLN, ZO1, AQP4, and AQP8 and to model the predictive power of these variables for expression level categories.

Differences in immunohistochemical scores for biomarkers (CLDN3, OCLN, ZO1, AQP4, and AQP8) at different postbiotic doses across each intestinal segment (duodenum, jejunum, ileum, cecum, colon, rectum) were evaluated using the Brown-Forsythe and Welch ANOVA tests, followed by a Dunnett’s T3 *post hoc* test. A * *p* < 0.05 was considered statistically significant.

## 3. Results

### 3.1. Postbiotic Characterization

The physicochemical and antioxidant characteristics of the postbiotic preparation are presented in Table 1. The pH of the preparation was acidic, indicating active metabolic fermentation. In addition, the total phenolic content and antioxidant activity values obtained from DPPH and ABTS assays demonstrated the presence of bioactive compounds in the postbiotic preparation. These findings confirm that the metabolites produced by *P. acidilactici* contribute to the biochemical composition of the obtained postbiotic product. The high bacterial cell density achieved during fermentation (approximately 9.0 log_10_ CFU/mL) may have contributed to the accumulation of microbial metabolites in the culture medium, thereby influencing the biochemical composition of the resulting postbiotic preparation. Overall, these results indicate that fermentation with *P. acidilactici* produced a metabolite-rich postbiotic fraction.

#### 3.1.1. Postbiotic Organic Acid Content

The analysis revealed that the postbiotic derived from *P. acidilactici* contained the highest amounts of citric acid (8.69 mg/mL) and lactic acid (6.42 mg/mL) (Figure S1, Table 2).

#### 3.1.2. Phenolic and Flavonoid Compound Content

The phenolic compound analysis revealed that the dominant compounds were naringin (13.62 mg/L), vanillin (8.85 mg/L), and chlorogenic acid (5.17 mg/L) (Figure S2, Table 3).

#### 3.1.3. Volatile Compounds

To characterize the chemical composition of the postbiotic preparation and identify volatile metabolites produced during fermentation by *P. acidilactici,* GC–MS analysis was performed. This analysis enabled the identification of various volatile bioactive compounds in the postbiotic fraction; the detected compounds and their relative abundances are presented in Table 4 and Figure S3. The most abundant compound was acetic acid (43.68%), followed by benzaldehyde (11.60%), butanal (3-methyl-), and pyrazine derivatives.

### 3.2. Histopathological Findings

**Figure 1:**
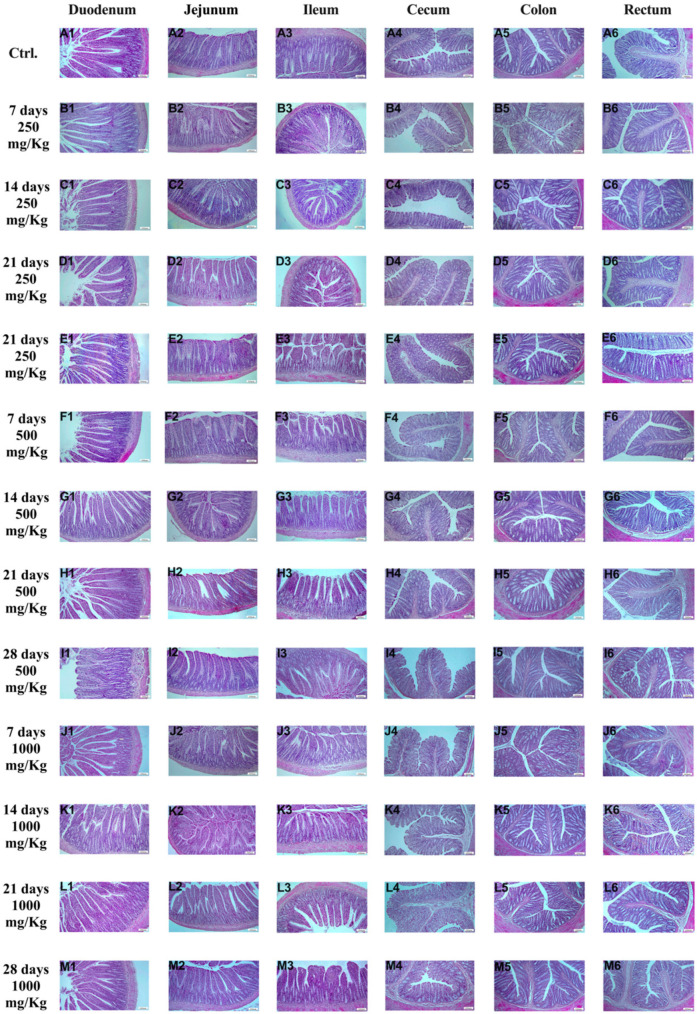
Histomorphological pictures of rat intestine segments after different treatments: (**A1**–**M1**) duodenum, (**A2**–**M2**) jejunum, (**A3**–**M3**) ileum, (**A4**–**M4**) cecum, (**A5**–**M5**) colon, and (**A6**–**M6**) rectum. HE, X100, Bar; 200 μm.

In the study, histopathological examination of different intestinal segments (duodenum, jejunum, ileum, cecum, colon, rectum) was conducted (Figure 1).

No pathological findings were found in the control group (Figure 1A1–A6) or in the treated groups: 7 days/250 mg/Kg (Figure 1B1–B6), 14 days/250 mg/Kg (Figure 1C1–C6), 21 days/250 mg/Kg (Figure 1D1–D6), 28 days/250 mg/Kg (Figure 1E1–E6), 7 days/500 mg/Kg (Figure 1F1–F6), 14 days/500 mg/Kg (Figure 1G1–G6), 21 days/500 mg/Kg (Figure 1H1–H6), 28 days/500 mg/Kg (Figure 1I1–I6), 7 days/1000 mg/Kg (Figure 1J1–J6), 14 days/1000 mg/Kg (Figure 1K1–K6), 21 days/1000 mg/Kg (Figure 1L1–L6) and 28 days/1000 mg/Kg (Figure 1M1–M6) groups.

### 3.3. Immunohistochemical Findings

For immunohistochemical analysis, the expression of CLDN3, OCLN, ZO1, AQP4, and AQP8 was examined in all intestinal segments.

**Figure 2:**
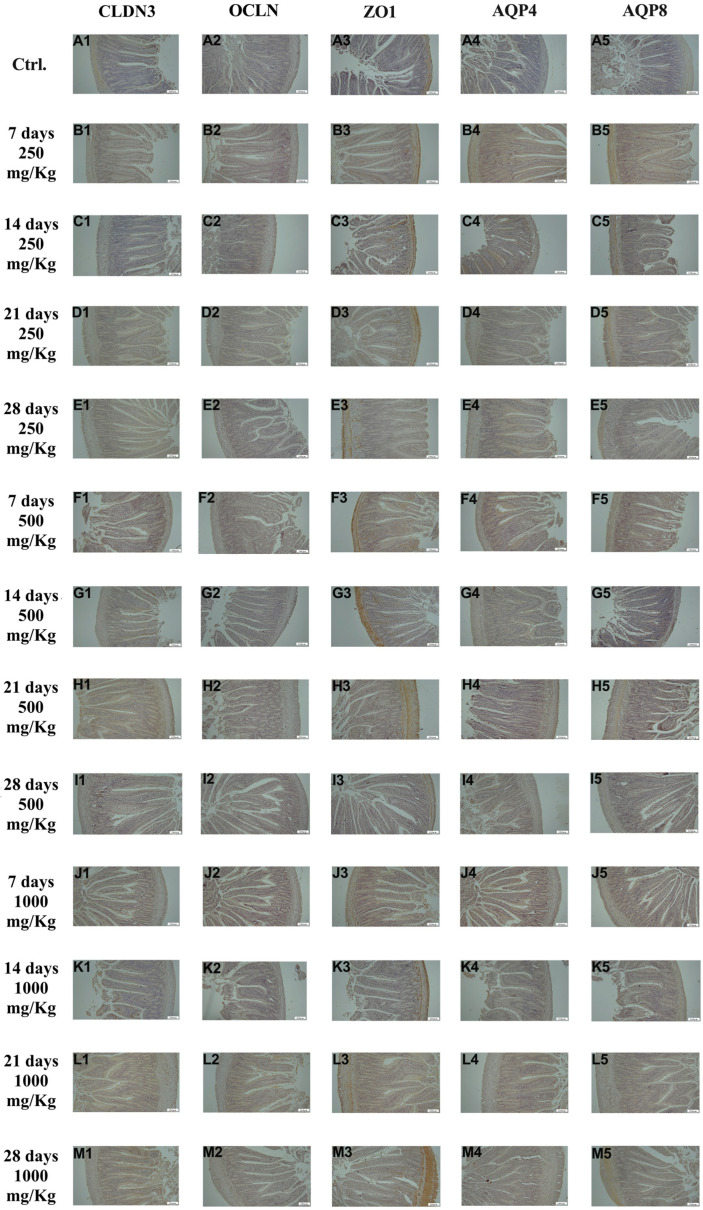
Immunohistochemical pictures of rat duodenum after different treatments: (**A1**–**M1**) CLDN3, (**A2**–**M2**) OCLN, (**A3**–**M3**) ZO1, (**A4**–**M4**) AQP4, and (**A5**–**M5**) AQP8. Varying levels of intestinal-specific biomarker expression in duodenum tissue. IHC, X100, Bar; 200 μm.

As shown in Figure 2, immunohistochemical examination of duodenal tissue revealed the expression of the biomarkers CLDN3, OCLN, ZO1, AQP4, and AQP8 in the control group (Figure 2A1–A5). The expression of these markers increased following administration of 250 mg/Kg postbiotic at days 7 (Figure 2B1–B5), 14 (Figure 2C1–C5), 21 (Figure 2D1–D5), and 28 (Figure 2E1–E5), regardless of the duration of administration. Similarly, administration of 500 mg/Kg postbiotic resulted in a further increase in the expression of CLDN3, OCLN, ZO1, AQP4, and AQP8 at days 7 (Figure 2F1–F5), 14 (Figure 2G1–G5), 21 (Figure 2H1–H5), and 28 (Figure 2I1–I5), compared to the 250 mg/Kg, regardless of treatment duration. At a dosage of 1000 mg/Kg, the expression levels of CLDN3, OCLN, ZO1, AQP4, and AQP8 were even higher at days 7 (Figure 2J1–J5), 14 (Figure 2K1–K5), 21 (Figure 2L1–L5), and 28 (Figure 2M1–M5) compared to the 500 mg/Kg groups, again regardless of the duration of administration. Postbiotic application increased the expression of CLDN3, OCLN, ZO1, AQP4, and AQP8 in duodenal tissue in a dose-dependent manner, with no significant effect of application time.

**Figure 3:**
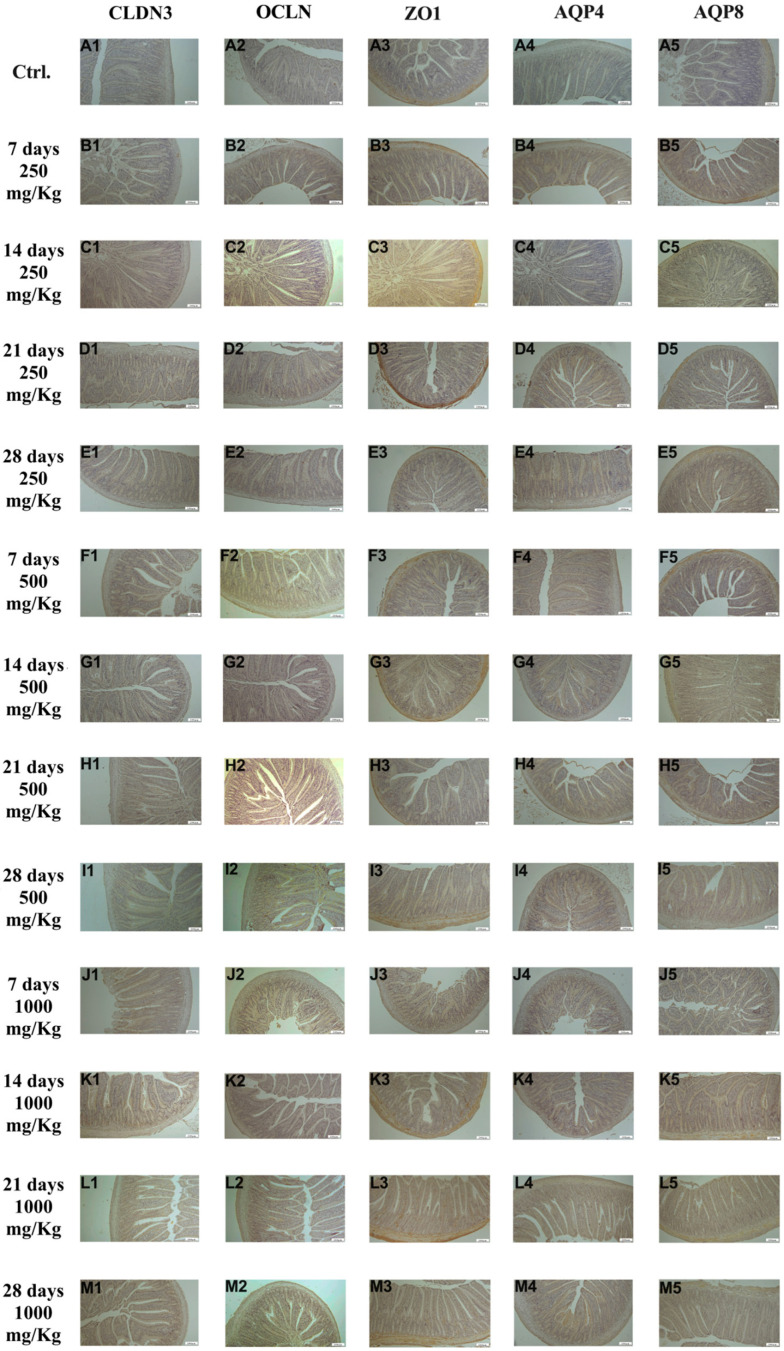
Immunohistochemical pictures of rat jejunum after different treatments: (**A1**–**M1**) CLDN3, (**A2**–**M2**) OCLN, (**A3**–**M3**) ZO1, (**A4**–**M4**) AQP4, and (**A5**–**M5**) AQP8. Varying levels of intestinal-specific biomarker expression in jejunal tissue. IHC, X100, Bar; 200 μm.

Immunohistochemical examination of jejunum tissue revealed the expression of the biomarkers CLDN3, OCLN, ZO1, AQP4, and AQP8 in the control group (Figure 3A1–A5). However, the expression these markers increased following administration of 250 mg/Kg postbiotic ad days 7 (Figure 3B1–B5), 14 (Figure 3C1–C5), 21 (Figure 3D1–D5), and 28 (Figure 3E1–E5), regardless of the duration of administration. Similarly, administration of 500 mg/Kg postbiotic resulted in a further increase in the expression of CLDN3, OCLN, ZO1, AQP4, and AQP8 at days 7 (Figure 3F1–F5), 14 (Figure 3G1–G5), 21 (Figure 3H1–H5), and 28 (Figure 3I1–I5), compared to the 250 mg/Kg groups, irrespective of treatment duration. At a dosage of 1000 mg/Kg, the expression levels of CLDN3, OCLN, ZO1, AQP4, and AQP8 were even higher ad days 7 (Figure 3J1–J5), 14 (Figure 3K1–K5), 21 (Figure 3L1–L5), and 28 (Figure 3M1–M5) compared to the 500 mg/Kg groups. Overall, postbiotic application increased the expression of CLDN3, OCLN, ZO1, AQP4, and AQP8 in jejunal tissue in a dose-dependent manner, with no significant effect of application time.

**Figure 4:**
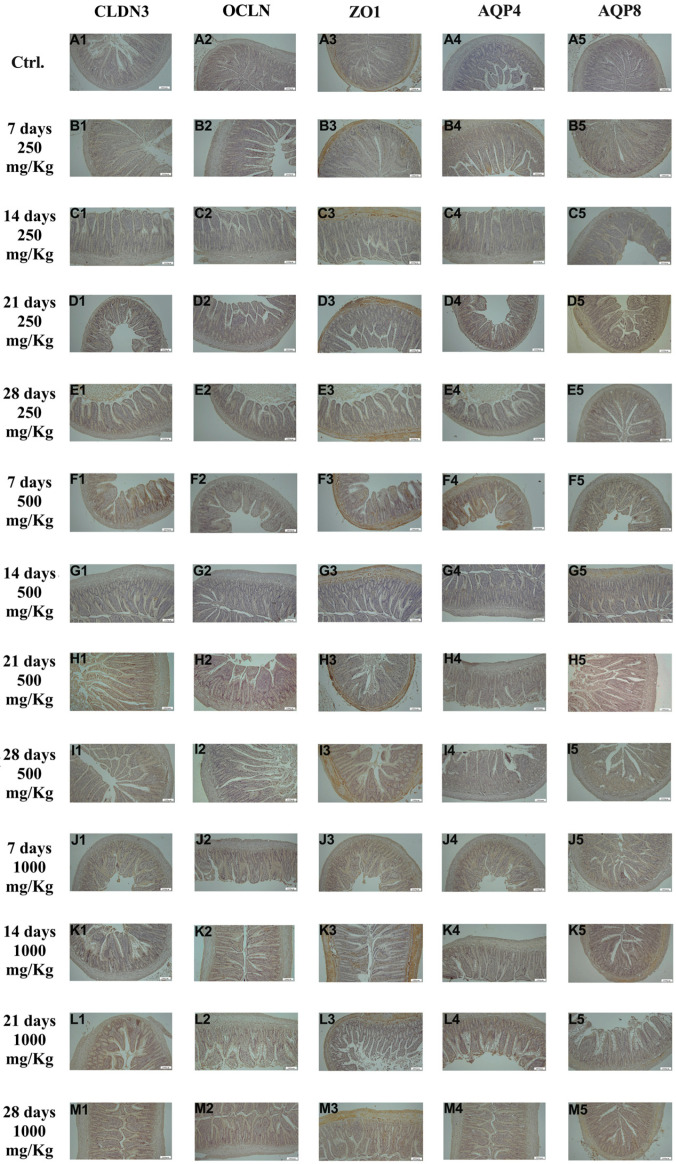
Immunohistochemical pictures of rat ileum after different treatments: (**A1**–**M1**) CLDN3, (**A2**–**M2**) OCLN, (**A3**–**M3**) ZO1, (**A4**–**M4**) AQP4, and (**A5**–**M5**) AQP8. Varying levels of intestinal-specific biomarker expression in ileum tissue. IHC, X100, Bar; 200 μm.

Immunohistochemical examination of ileal tissue revealed the expression of the biomarkers CLDN3, OCLN, ZO1, AQP4, and AQP8 in the control group (Figure 4A1–A5). However, the expression of these markers increased following administration of 250 mg/Kg postbiotic at days 7 (Figure 4B1–B5), 14 (Figure 4C1–C5), 21 (Figure 4D1–D5), and 28 (Figure 4E1–E5), regardless of the duration of administration. Similarly, administration of 500 mg/Kg postbiotic resulted in a further increase in the expression of CLDN3, OCLN, ZO1, AQP4, and AQP8 at days 7 (Figure 4F1–F5), 14 (Figure 4G1–G5), 21 (Figure 4H1–H5), and 28 (Figure 4I1–I5), compared to the 250 mg/Kg groups, irrespective of treatment duration. At a dosage of 1000 mg/Kg, the expression levels of CLDN3, OCLN, ZO1, AQP4, and AQP8 were even at days 7 (Figure 4J1–J5), 14 (Figure 4K1–K5), 21 (Figure 4L1–L5), and 28 (Figure 4M1–M5) compared to the 500 mg/Kg groups, again independent of the duration of administration. Overall, postbiotic application increased the expression of CLDN3, OCLN, ZO1, AQP4, and AQP8 in a dose-dependent manner in ileal tissue, with no significant effect of application time.

**Figure 5:**
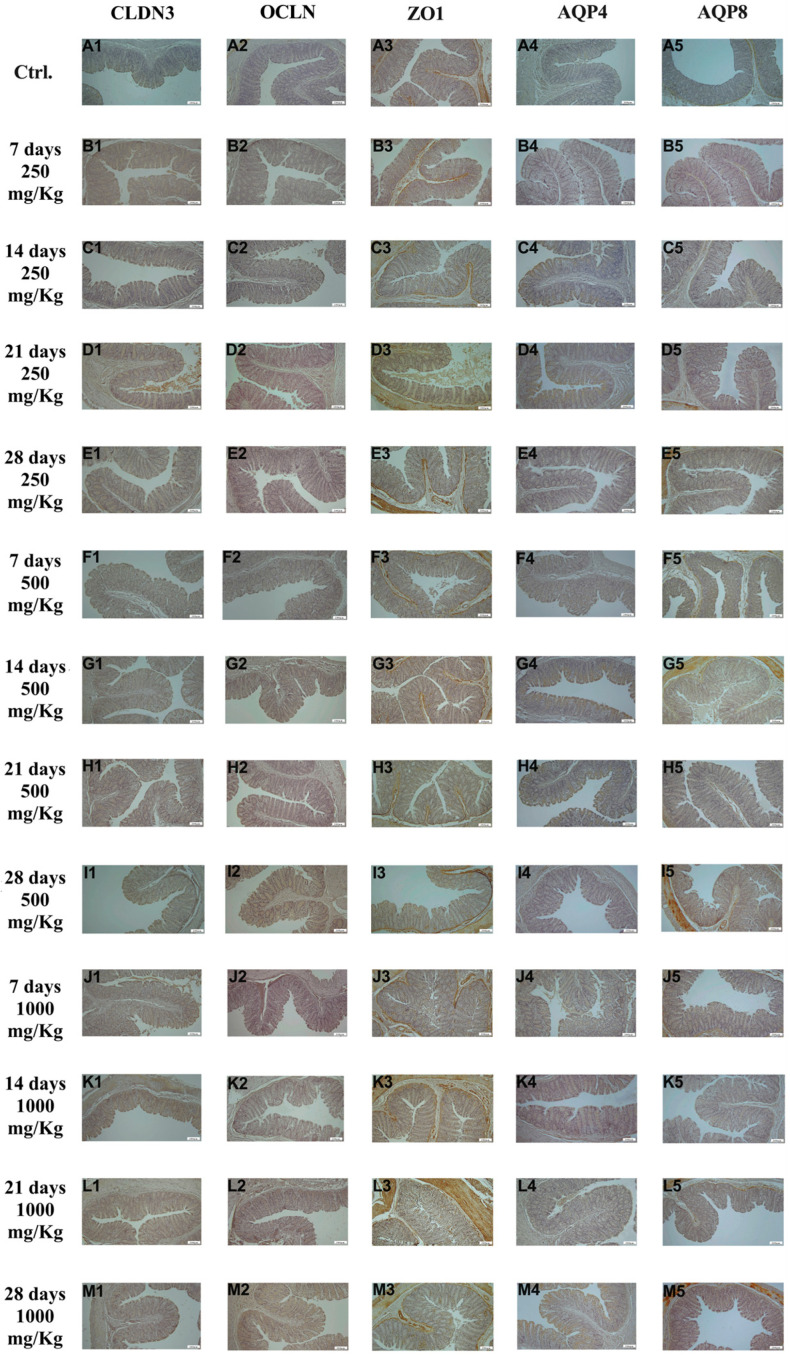
Immunohistochemical pictures of rat cecum after different treatments: (**A1**–**M1**) CLDN3, (**A2**–**M2**) OCLN, (**A3**–**M3**) ZO1, (**A4**–**M4**) AQP4, and (**A5**–**M5**) AQP8. IHC, X100, Bar; 200 μm.

Immunohistochemical examination of cecal tissue revealed the expression of the biomarkers CLDN3, OCLN, ZO1, AQP4, and AQP8 in the control group (Figure 5A1–A5). However, the expression of these markers increased following administration of 250 mg/Kg postbiotic at days 7 (Figure 5B1–B5), 14 (Figure 5C1–C5), 21 (Figure 5D1–D5), and 28 (Figure 5E1–E5), regardless of the duration of administration. Similarly, administration of 500 mg/Kg postbiotic resulted in a further increase in the expression of CLDN3, OCLN, ZO1, AQP4, and AQP8 at days 7 (Figure 5F1–F5), 14 (Figure 5G1–G5), 21 (Figure 5H1–H5), and 28 (Figure 5I1–I5), compared to the 250 mg/Kg groups, irrespective of duration of administration. At a dosage of 1000 mg/Kg, the expression levels of CLDN3, OCLN, ZO1, AQP4, and AQP8 were even higher at days 7 (Figure 5J1–J5), 14 (Figure 5K1–K5), 21 (Figure 5L1–L5), and 28 (Figure 5M1–M5) compared to the 500 mg/Kg groups, again independent of the duration of administration. Overall, postbiotic application increased the expression of CLDN3, OCLN, ZO1, AQP4, and AQP8 in cecal tissue in a dose-dependent manner, with no significant effect of application time.

**Figure 6:**
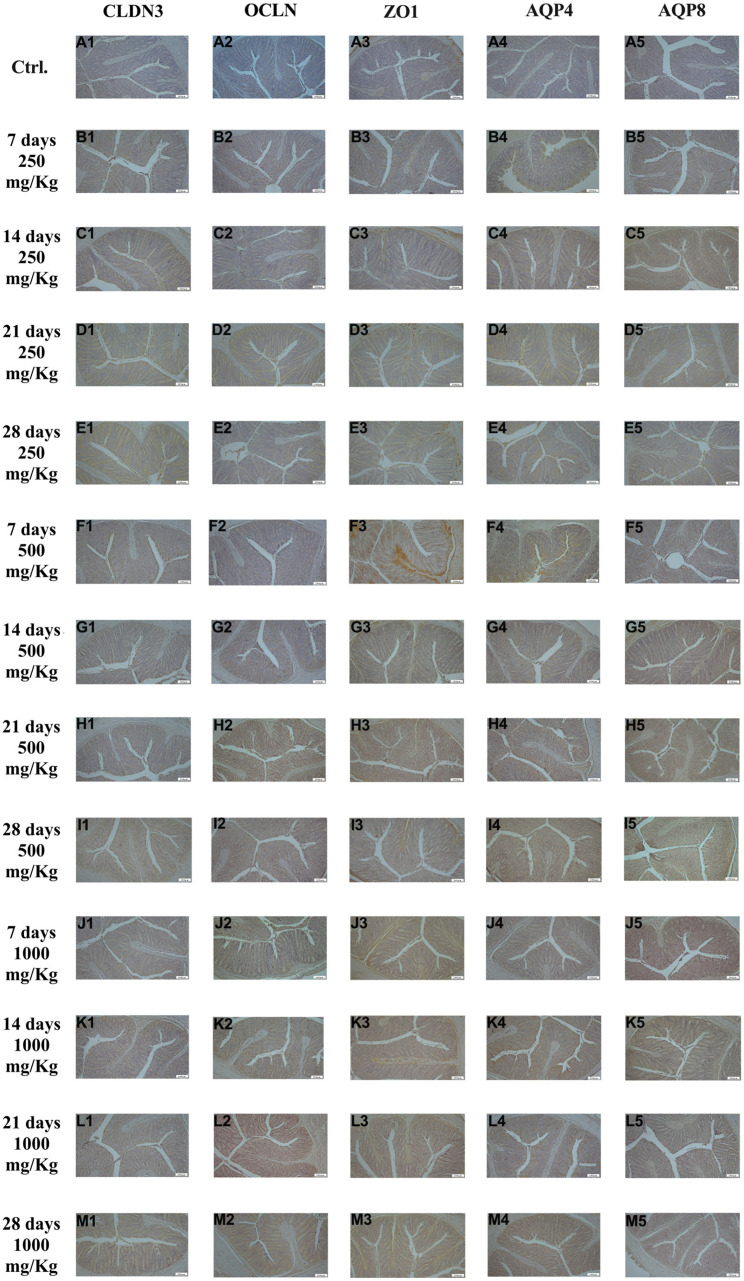
Immunohistochemical pictures of rat colon after different treatments: (**A1**–**M1**) CLDN3, (**A2**–**M2**) OCLN, (**A3**–**M3**) ZO1, (**A4**–**M4**) AQP4, and (**A5**–**M5**) AQP8. IHC, X100, Bar; 200 μm.

Immunohistochemical examination of colon tissue revealed expression of the biomarkers CLDN3, OCLN, ZO1, AQP4, and AQP8 in the control group (Figure 6A1–A5). However, the expression of these markers increased following administration of 250 mg/Kg postbiotic at days 7 (Figure 6B1–B5), 14 (Figure 6C1–C5), 21 (Figure 6D1–D5), and 28 (Figure 6E1–E5), regardless of the duration of administration. Similarly, administration of 500 mg/Kg postbiotic resulted in a further increase in the expression of CLDN3, OCLN, ZO1, AQP4, and AQP-8 at days 7 (Figure 6F1–F5), 14 (Figure 6G1–G5), 21 (Figure 6H1–H5), and 28 (Figure 6I1–I5), compared to the 250 mg/Kg groups, irrespective of treatment duration. At a dosage of 1000 mg/Kg, the expression levels of CLDN3, OCLN, ZO1, AQP4, and AQP8 were even higher at days 7 (Figure 6J1–J5), 14 (Figure 6K1–K5), 21 (Figure 6L1–L5), and 28 (Figure 6M1–M5) compared to the 500 mg/Kg groups, again independent of the duration of administration. Overall, postbiotic application increased the expression of CLDN3, OCLN, ZO1, AQP4, and AQP8 in colon tissue in a dose-dependent manner, with no significant effect of application time.

**Figure 7:**
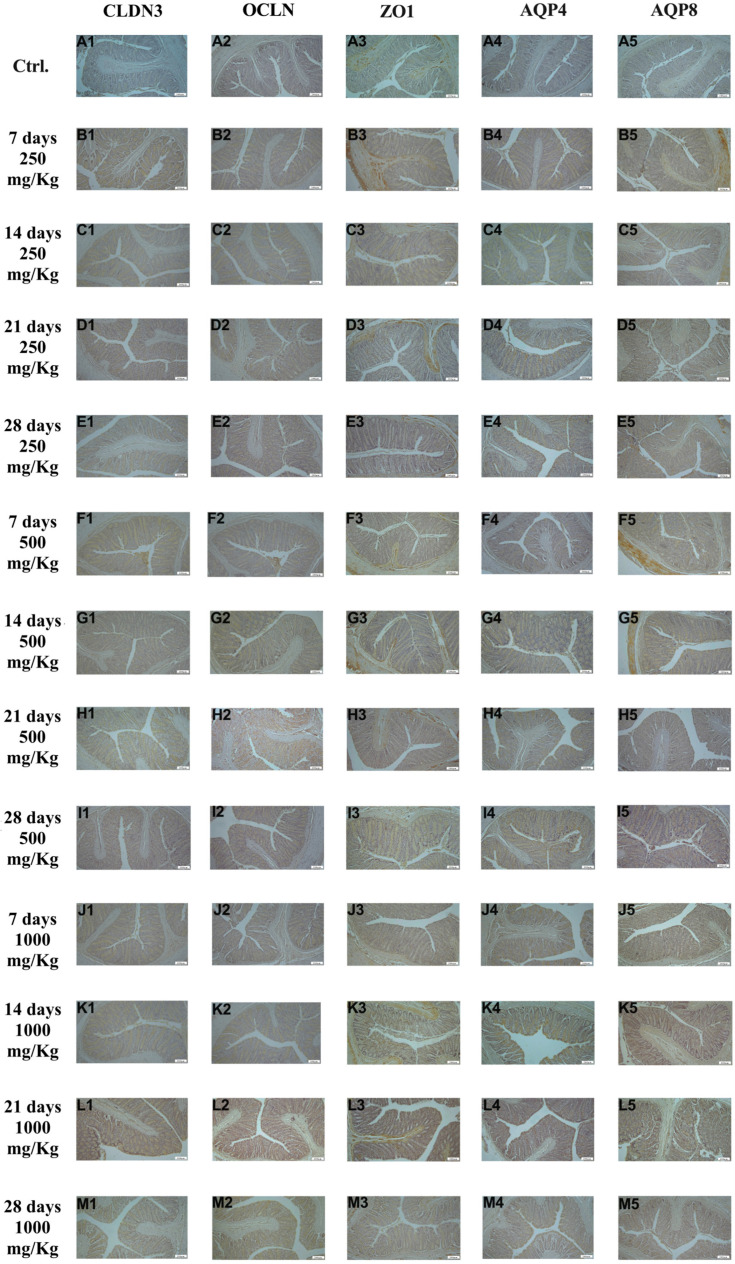
Immunohistochemical pictures of rat rectum after different treatments: (**A1**–**M1**) CLDN3, (**A2**–**M2**) OCLN, (**A3**–**M3**) ZO1, (**A4**–**M4**) AQP4, and (**A5**–**M5**) AQP8. IHC, X100, Bar; 200 μm.

Immunohistochemical examination of rectal tissue revealed the expression of the biomarkers CLDN3, OCLN, ZO1, AQP4, and AQP8 in the control group (Figure 7A1–A5). However, the expression of these markers increased following administration of 250 mg/Kg postbiotic at days 7 (Figure 7B1–B5), 14 (Figure 7C1–C5), 21 (Figure 7D1–D5), and 28 (Figure 7E1–E5), regardless of the duration of administration. Similarly, administration of 500 mg/Kg postbiotic resulted in a further increase in the expression of CLDN3, OCLN, ZO1, AQP4, and AQP8 at days 7 (Figure 7F1–F5), 14 (Figure 7G1–G5), 21 (Figure 7H1–H5), and 28 (Figure 7I1–I5), compared to the 250 mg/Kg groups, irrespective of treatment duration. At a dosage of 1000 mg/Kg, the expression levels of CLDN3, OCLN, ZO1, AQP4, and AQP8 were even higher at days 7 (Figure 7J1–J5), 14 (Figure 7K1–K5), 21 (Figure 7L1–L5), and 28 (Figure 7M1–M5) compared to the 500 mg/Kg groups, again independent of the duration of administration. Overall, postbiotic application increased the expression of CLDN3, OCLN, ZO1, AQP4, and AQP8 in rectal tissue in a dose-dependent manner, with no significant effect of application time.

**Figure 8:**
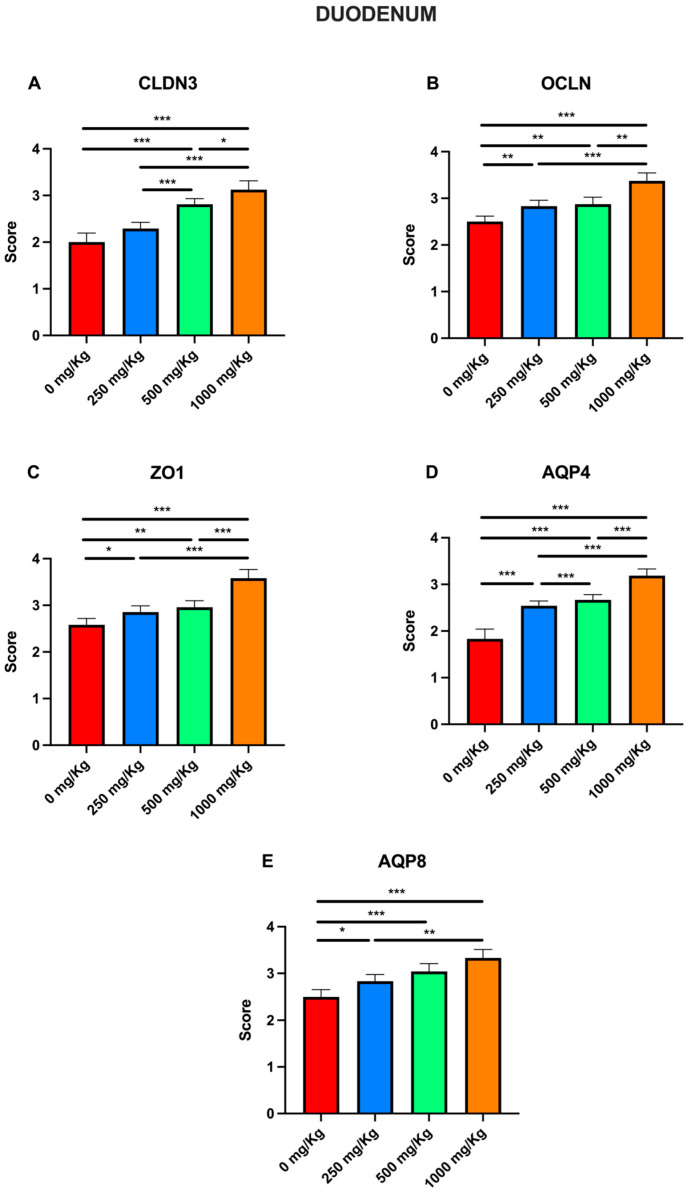
Immunohistochemical evaluation scores for (**A**) CLDN3, (**B**) OCLN, (**C)** ZO1, (**D**) AQP4, and (**E**) AQP8 at different postbiotic doses in the duodenum (* *p* < 0.05, ** *p* < 0.01, *** *p* < 0.001).

In Figure 8, the immunohistochemical evaluation scores for CLDN3, OCLN, ZO1, AQP4, and AQP8 at different postbiotic dosages in the duodenum are shown. Compared to the control, postbiotic administration caused a significant increase (*p* < 0.001) in CLDN3 scores in all groups except the 250 mg/Kg group (Figure 8A), rising from 2 ± 0.07 to 2.81 ± 0.49 at 500 mg/Kg, and to 3.12 ± 0.07 at 1000 mg/Kg. A similar trend was observed when comparing the 250 mg/Kg group with the 500 and 1000 mg/Kg groups (*p* < 0.001). The level of significance decreased when comparing the 500 mg/Kg group with the 1000 mg/Kg group (*p* < 0.05). For OCLN, postbiotic administration caused a significant increase in scores in all groups with respect to the control (Figure 8B), from 2.5 ± 0.48 to 2.83 ± 0.51 at 250 mg/Kg (*p* < 0.01), 2.87 ± 0.06 at 500 mg/Kg (*p* < 0.01), and 3.37 ± 0.07 at 1000 mg/Kg (*p* < 0.001). Significant increases were also observed when comparing the 250 mg/Kg group with the 1000 mg/Kg group (*p* < 0.001) and the 500 mg/Kg group with the 1000 mg/Kg group (*p* < 0.01).

Similarly, for ZO1, postbiotic administration caused a significant increase in scores in all groups compared with the control (Figure 8C), from 2.58 ± 0.55 to 2.85 ± 0.54 at 250 mg/Kg (*p* < 0.05), 2.95 ± 0.06 at 500 mg/Kg (*p* < 0.01) and 3.58 ± 0.07 at 1000 mg/Kg (*p* < 0.001). Significant increases (*p* < 0.001) were also observed when comparing the 250 mg/Kg group with the 1000 mg/Kg group and the 500 mg/Kg group with the 1000 mg/Kg group. Postbiotic administration also caused a significant increase (*p* < 0.001) in AQP4 scores in all groups with respect to the control (Figure 8D), from 1.83 ± 0.08 to 2.54 ± 0.04 at 250 mg/Kg, 2.66 ± 0.04 at 500 mg/Kg, and 3.18 ± 0.05 at 1000 mg/Kg. A similar trend was observed when comparing the 250 mg/Kg group to the 500 and 1000 mg/Kg groups (*p* < 0.001). For AQP8, postbiotic administration also resulted in a significant increase in scores in all groups compared with the control (Figure 8E), from 2.5 ± 0.06 to 2.83 ± 0.05 at 250 mg/Kg (*p* < 0.05), 3.04 ± 0.06 at 500 mg/Kg (*p* < 0.001) and 3.33 ± 0.07 at 1000 mg/Kg (*p* < 0.001). A similar trend was observed when comparing the 250 mg/kg group with the 1000 mg/Kg group (*p* < 0.01).

**Figure 9:**
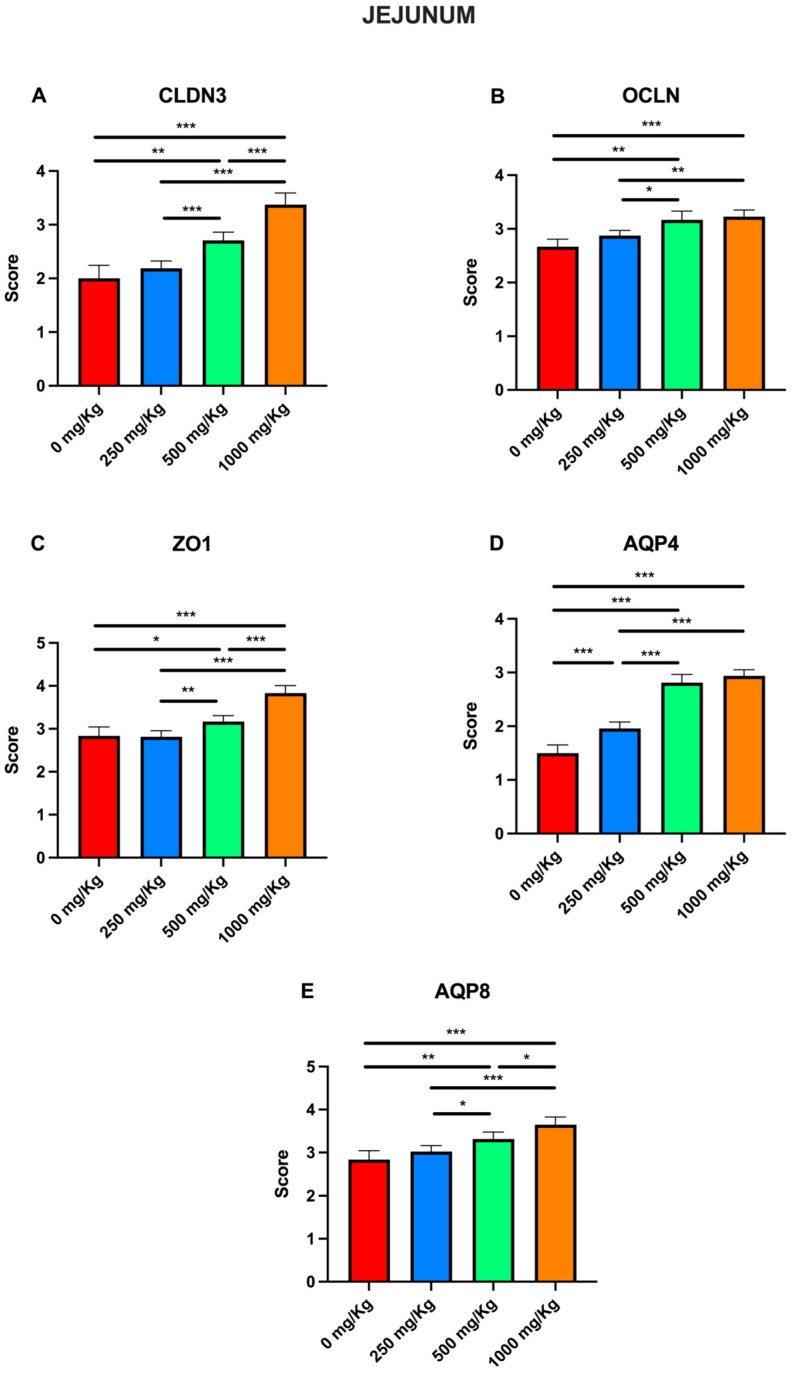
Immunohistochemical evaluation scores for (**A**) CLDN3, (**B**) OCLN, (**C**) ZO1, (**D**) AQP4, and (**E**) AQP8 at different postbiotic doses in the jejunum (* *p* < 0.05, ** *p* < 0.01, *** *p* < 0.001).

Immunohistochemical evaluation scores for CLDN3, OCLN, ZO1, AQP4, and AQP8 at different postbiotic dosages were also analyzed for the jejunum (Figure 9). With the only exception of the 250 mg/Kg group, postbiotic administration caused a significant increase in CLDN3 scores in all groups compared with the control (Figure 9A), rising from 2 ± 0.1 to 2.70 ± 0.06 at 500 mg/Kg (*p* < 0.01) and to 3.37 ± 0.08 at 1000 mg/Kg (*p* < 0.001). A significant increase was also observed when comparing the 250 mg/Kg group with the 500 mg/Kg group (from 2.18 ± 0.05 to 2.70 ± 0.06; *p* < 0.001) and with the 1000 mg/Kg group (*p* < 0.001), as well as between the 500 and the 1000 mg/Kg groups (*p* < 0.05). Similarly, postbiotic administration caused a significant increase in OCLN scores in all groups with respect to the control (Figure 9B), except for the 250 mg/Kg group, increasing from 2.66 ± 0.05 to 3.16 ± 0.06 at 500 mg/Kg (*p* < 0.01) and to 3.22 ± 0.05 at 1000 mg/Kg (*p* < 0.001). Moreover, significant increases were observed when comparing the 250 mg/Kg group with the 500 mg/Kg group (from 2.66 ± 0.05 to 2.87 ± 0.03; *p* < 0.05) and with the 1000 mg/Kg group (*p* < 0.01). 

Similarly, for ZO1, postbiotic administration caused a significant increase in scores in all groups compared with the control (Figure 9C), except for the 250 mg/Kg group, rising from 2.83 ± 0.08 to 3.16 ± 0.05 at 500 mg/Kg (*p* < 0.05) and to 3.83 ± 0.07 at 1000 mg/Kg (*p* < 0.001). When comparing the 250 mg/Kg group with the 500 mg/Kg group, the score significantly increased from 2.81 ± 0.05 to 3.16 ± 0.06 (*p* < 0.01), as well as when comparing the 250 mg/Kg group to the 1000 mg/Kg group (*p* < 0.001) and the 500 mg/Kg group to the 1000 mg/Kg group (*p* < 0.001). For AQP4, postbiotic administration caused a significant increase (*p* < 0.001) in scores in all groups compared with the control (Figure 9D), from 1.5 ± 0.06 to 1.95 ± 0.04 at 250 mg/Kg, 2.81 ± 0.06 at 500 mg/Kg, and 2.93 ± 0.04 at 1000 mg/Kg. A similar trend was observed when comparing the 250 mg/kg, 500 mg/kg, and 1000 mg/Kg groups (*p* < 0.001). For AQP8, postbiotic administration caused a significant increase in scores in all groups compared with the control (Figure 9E), except for the 250 mg/kg group, increasing from 2.83 ± 0.08 to 3.31 ± 0.06 at 500 mg/Kg (*p* < 0.01) and to 3.64 ± 0.07 at 1000 mg/Kg (*p* < 0.001). A similar trend was also observed when comparing the 250 mg/Kg group with the 500 mg/Kg group (from 3.02 ± 0.05 to 3.31 ± 0.06; *p* < 0.05), and with the 1000 mg/Kg group (*p* < 0.001), as well as between the 500 and 1000 mg/Kg groups (*p* < 0.05).

**Figure 10:**
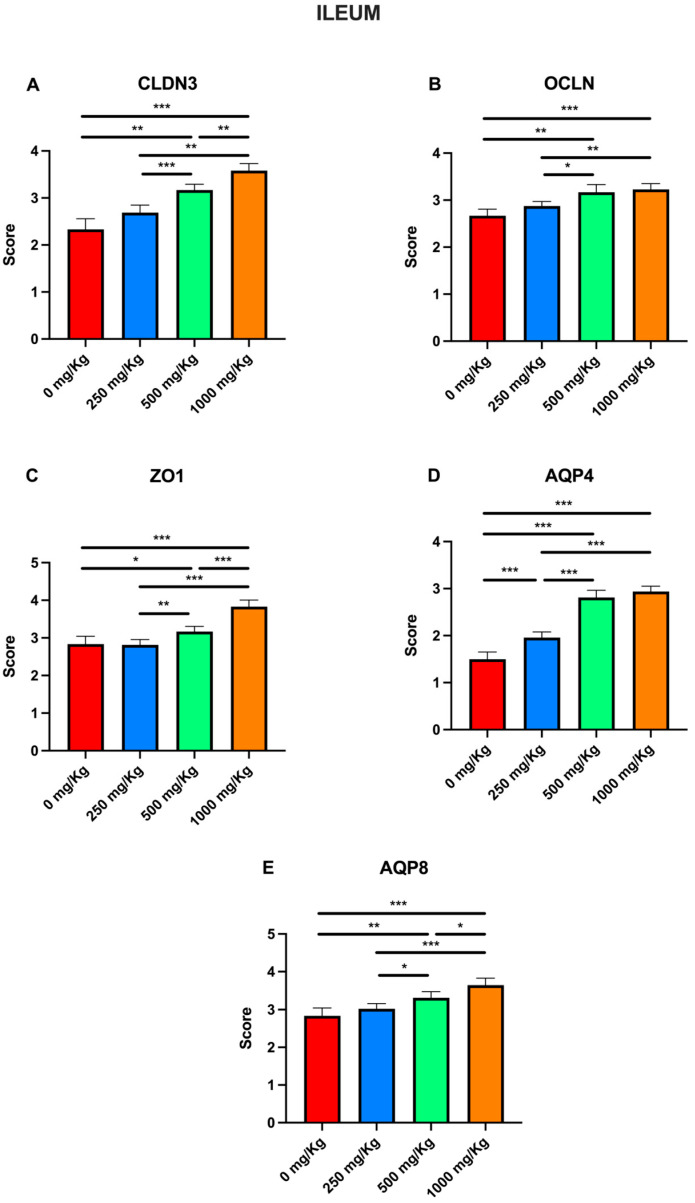
Immunohistochemical evaluation scores for (**A**) CLDN3, (**B**) OCLN, (**C**) ZO1, (**D**) AQP4, and (**E**) AQP8 at different postbiotic doses in the ileum (* *p* < 0.05, ** *p* < 0.01, *** *p* < 0.001).

Immunohistochemical evaluation scores for CLDN3, OCLN, ZO1, AQP4, and AQP8 at different postbiotic dosages were also analyzed in the ileum (Figure 10). Compared to the control, postbiotic administration significantly increased CLDN3 scores in the 500 and 1000 mg/Kg groups (*p* < 0.001; Figure 10A), rising from 2.33 ± 0.09 to 3.16 ± 0.05 and to 3.58 ± 0.06, respectively. Significant increases were also observed when comparing the 250 mg/Kg group (2.68 ± 0.06) to both the 500 mg/Kg (3.16 ± 0.05, *p* < 0.001) and 1000 mg/Kg groups (3.58 ± 0.06, *p* < 0.01), as well as between the 500 and the 1000 mg/Kg groups (*p* < 0.01). Similarly to CLDN3, postbiotic administration significantly increased OCLN scores relative to the control only in the 500 and 1000 mg/Kg groups (Figure 10B), increasing from 2.58 ± 0.05 to 3 ± 0.06 (*p* < 0.01) and 3.66 ± 0.07 (*p* < 0.001), respectively. Furthermore, scores significantly increased when comparing the 250 mg/Kg group to the 1000 mg/Kg group (from 2.77 ± 0.03 to 3.66 ± 0.07; *p* < 0.001), and when comparing the 500 mg/Kg group to the 1000 mg/Kg group (*p* < 0.001). Regarding ZO1, postbiotic treatment significantly increased scores in all groups except for the 250 mg/Kg group relative to the control (Figure 10C), with values rising from 2.5 ± 0.06 to 2.87 ± 0.06 at 500 mg/Kg (*p* < 0.01) and 3.45 ± 0.07 at 1000 mg/Kg (*p* < 0.001). Significant increases were also noted when comparing the 250 mg/Kg group (2.58 ± 0.04) to the 500 (2.87 ± 0.06, *p* < 0.05) and 1000 mg/Kg groups (3.45 ± 0.07, *p* < 0.001). A similar trend was observed between the 500 and 1000 mg/kg groups (*p* < 0.001). For AQP4, postbiotic administration significantly increased scores in all groups except the 250 mg/Kg group (Figure 10D), rising from 2.16 ± 0.08 to 2.62 ± 0.04 at 500 mg/Kg (*p* < 0.01) and 3.22 ± 0.06 at 1000 mg/Kg (*p* < 0.001), as well as between the 500 and the 1000 mg/kg group (*p* < 0.001). Postbiotic administration caused a significant increase in AQP8 scores in the 500 and 1000 mg/kg groups relative to the control (Figure 10E), from 2.41 ± 0.07 to 2.97 ± 0.05 (*p* < 0.01) and 3.29 ± 0.07 (*p* < 0.001), respectively. Comparison showed significant increased from the 250 mg/Kg (2.64 ± 0.05) to both the 500 mg/Kg group (2.97 ± 0.05, *p* < 0.01) and 1000 mg/Kg groups (3.29 ± 0.07, *p* < 0.001), as well as between the 500 to the 1000 mg/Kg groups (*p* < 0.05).

**Figure 11:**
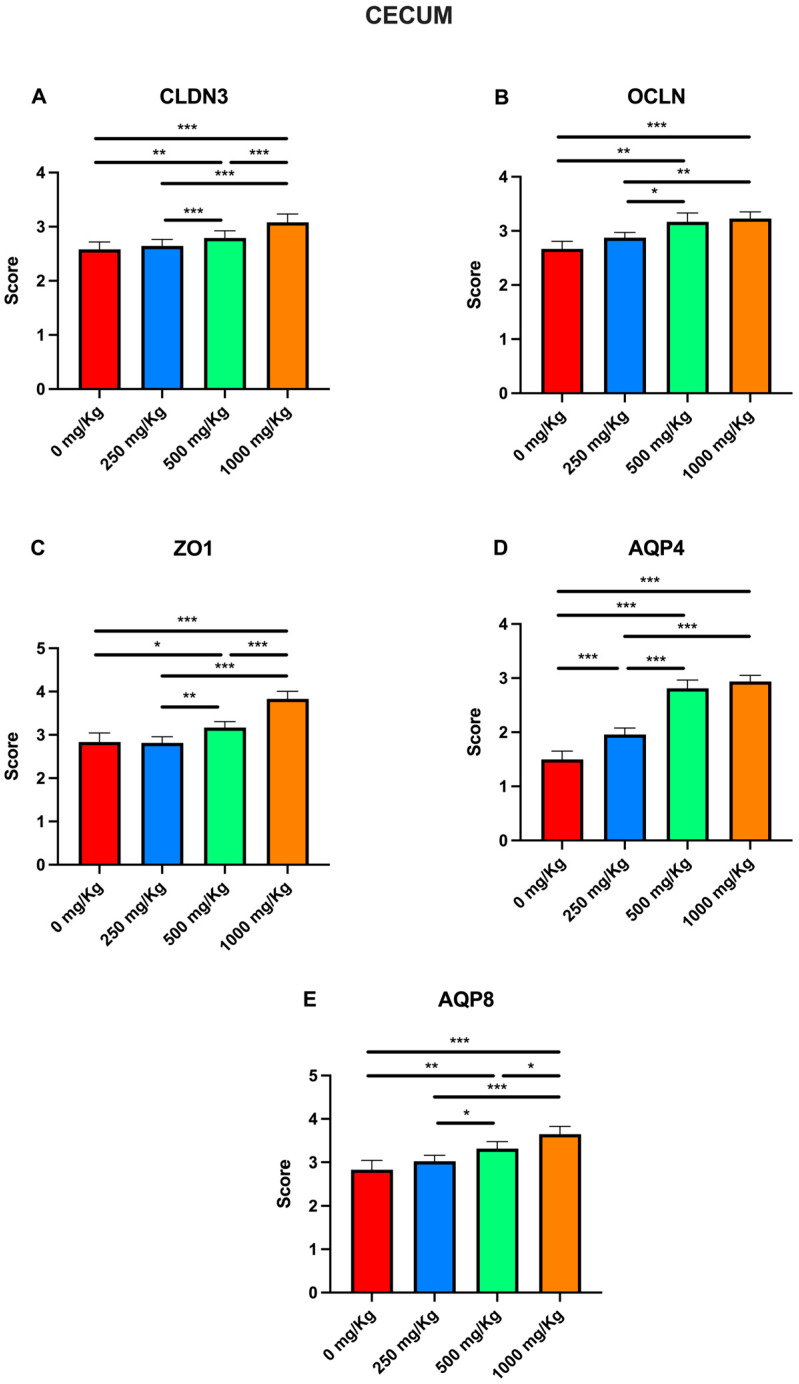
Immunohistochemical evaluation scores for (**A**) CLDN3, (**B**) OCLN, (**C**) ZO1, (**D**) AQP4, and (**E**) AQP8 at different postbiotic doses in the cecum (* *p* < 0.05, ** *p* < 0.01, *** *p* < 0.001).

Immunohistochemical evaluation scores for these markers were also analyzed in the cecum (Figure 11). Compared to the control, postbiotic administration significantly increased CLDN3 scores only in the 1000 mg/Kg group (2.58 ± 0.05 vs. 3.08 ± 0.06; *p* < 0.001; Figure 11A). Significant increases were also observed when comparing the 1000 mg/kg group to both the 250 mg/Kg group (2.64 ± 0.04 to 3.08 ± 0.06; *p* < 0.01), and the 500 mg/Kg group (2.79 ± 0.05 to 3.08 ± 0.06; *p* < 0.001). Consistent with the CLDN3 results, postbiotic administration caused a significant increase OCLN scores relative to the control in all except for the 250 mg/Kg group (Figure 11B), rising from 2 ± 0.1 to 2.47 ± 0.11 at 500 mg/Kg (*p* < 0.05) and 2.79 ± 0.06 at 1000 mg/Kg (*p* < 0.001). Furthermore, significant increases were noted when comparing the 250 mg/Kg group to the 1000 mg/Kg group (2.29 ± 0.04 to 2.79 ± 0.06; *p* < 0.001), and between the 500 and 1000 mg/Kg groups (*p* < 0.05). 

Similarly, postbiotic administration significantly increased ZO1 scores in all groups except for the 250 mg/Kg group (Figure 11C), with values increasing from 2.41 ± 0.07 to 2.72 ± 0.06 at 500 mg/Kg (*p* < 0.05) and 2.97 ± 0.06 at 1000 mg/Kg (*p* < 0.01). A significant increase was also observed between the 250 mg/Kg group and the 1000 mg/kg groups (2.54 ± 0.04 to 2.97 ± 0.06; *p* < 0.01). For AQP4, a significant increase was observed in the 1000 mg/Kg group with respect to the control (from 2.66 ± 0.09 to 3.12 ± 0.06; *p* < 0.05; Figure 11D), and between the 250 and the 1000 mg/Kg groups (2.77 ± 0.05 to 3.12 ± 0.06; *p* < 0.05). Regarding AQP8, scores significant increased in all groups with respect to the control except for the 250 mg/Kg group (Figure 11E), rising from 2.16 ± 0.08 to 2.75 ± 0.05 at 500 mg/Kg (*p* < 0.01) and 3 ± 0.06 at 1000 mg/Kg (*p* < 0.001). Similar trends were observed when comparing the 250 mg/Kg group (2.45 ± 0.06) to the 500 mg/Kg (*p* < 0.05) and 1000 mg/Kg groups (*p* < 0.001).

**Figure 12:**
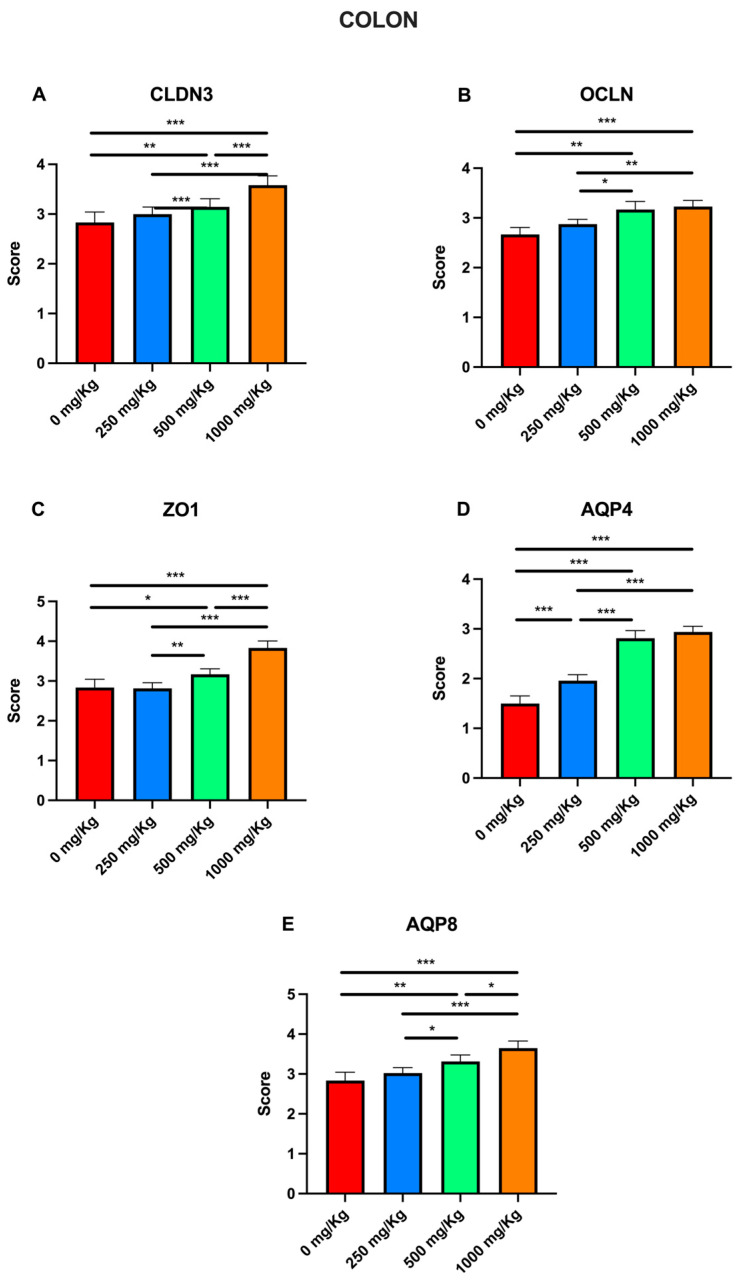
Immunohistochemical evaluation scores for (**A**) CLDN3, (**B**) OCLN, (**C**) ZO1, (**D**) AQP4, and (**E**) AQP8 at different postbiotic doses in the colon (* *p* < 0.05, ** *p* < 0.01, *** *p* < 0.001).

Finally, immunohistochemical evaluation scores for CLDN3, OCLN, ZO1, AQP4, and AQP8 across different postbiotic dosages were also analyzed for the colon (Figure 12). Compared with the control, postbiotic administration significantly increased the CLDN3 score only in the 1000 mg/Kg group (rising from 2.83 ± 0.08 to 3.58 ± 0.07; *p* < 0.001; Figure 12A). Significant increases were also observed when comparing the 1000 mg/Kg group to the 250 mg/Kg group (3 ± 0.05 to 3.58 ± 0.07; *p* < 0.001), and the 500 mg/Kg group (3.14 ± 0.06 to 3.58 ± 0.07; *p* < 0.01). Consistent with the CLDN3 findings, postbiotic administration significantly increased OCLN scores in all treatment groups relative to the control (Figure 12B), with values rising from 2.83 ± 0.08 to 3 ± 0.05 at 250 mg/Kg (*p* < 0.05), 3.14 ± 0.06 at 500 mg/Kg (*p* < 0.001) and 3.58 ± 0.07 at 1000 mg/Kg (*p* < 0.001). 

Furthermore, significant increases were noted when comparing the 250 mg/Kg group with both the 500 mg/Kg group (*p* < 0.01) and 1000 mg/Kg group (*p* < 0.001). Regarding ZO1, postbiotic treatment significantly increased scores in all groups except the 250 mg/Kg group (Figure 12C), rising from 2.16 ± 0.08 to 2.60 ± 0.05 at 500 mg/Kg (*p* < 0.05) and 2.95 ± 0.05 at 1000 mg/Kg (*p* < 0.001). Significant differences were also observed between the 1000 mg/Kg group and both the 250 mg/Kg (2.43 ± 0.04 to 2.95 ± 0.05; *p* < 0.001) and 500 mg/Kg groups (*p* < 0.01). For AQP4, a significant increase was observed in the 1000 mg/Kg group compared to both the control (from 2.91 ± 0.08 to 3.37 ± 0.06; *p* < 0.05; Figure 12D) and the 250 mg/Kg group (3.02 ± 0.06 to 3.37 ± 0.06; *p* < 0.05). For AQP8, scores significantly increased in the 500 and 1000 mg/Kg groups relative to the control (Figure 12E), rising from 1.83 ± 0.08 to 2.45 ± 0.04 (*p* < 0.01) and 2.68 ± 0.05 (*p* < 0.001), respectively. Similar trends were observed when comparing the 250 mg/Kg group (2.14 ± 0.05) with the 500 mg/Kg (*p* < 0.01) and 1000 mg/Kg groups (2.68 ± 0.06; *p* < 0.001).

**Figure 13:**
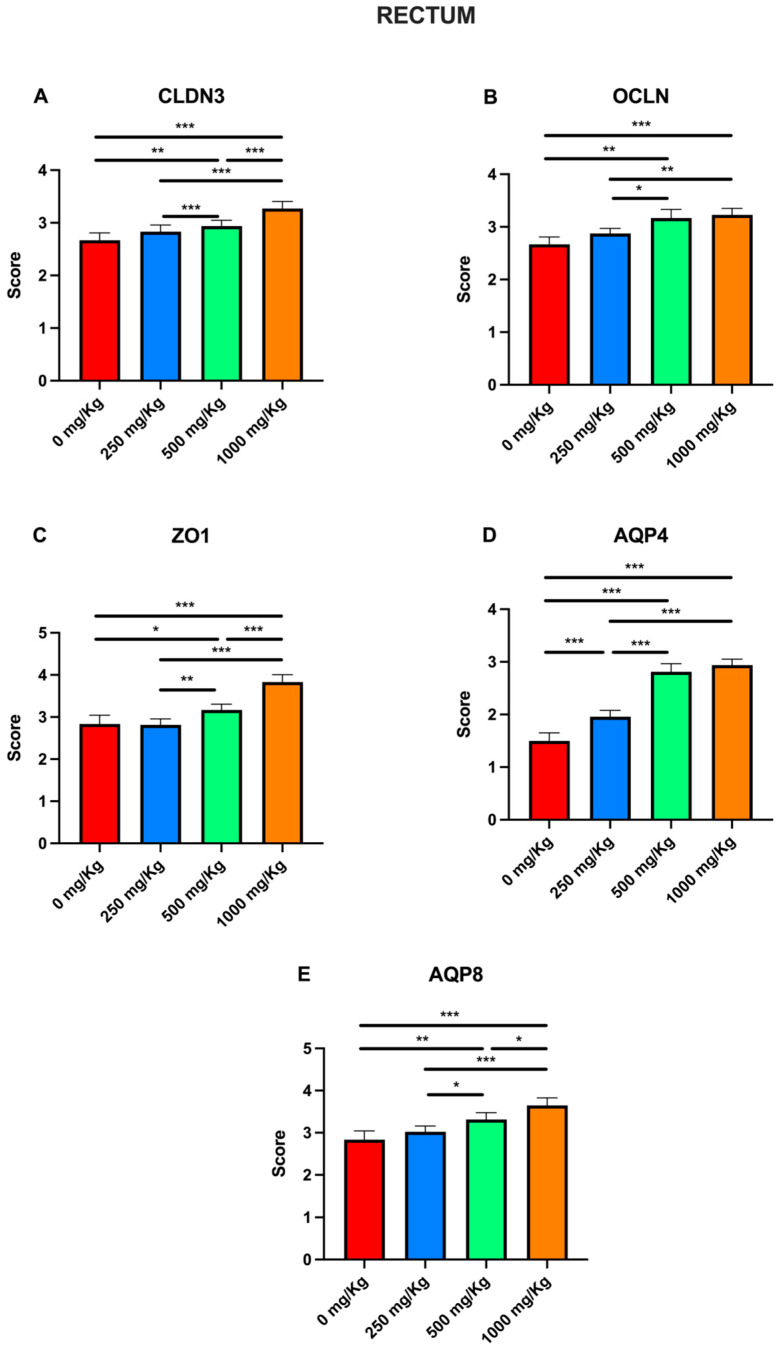
Immunohistochemical evaluation scores for (**A**) CLDN3, (**B**) OCLN, (**C**) ZO1, (**D**) AQP4, and (**E**) AQP8 at different postbiotic doses in the rectum (* *p* < 0.05, ** *p* < 0.01, *** *p* < 0.001).

Finally, immunohistochemical evaluation scores for CLDN3, OCLN, ZO1, AQP4, and AQP8 across different postbiotic dosages were also analyzed for the rectum (Figure 13).

Compared to the control, postbiotic administration significantly increased CLDN3 scores in the 500 and 1000 mg/Kg groups (Figure 13A), rising from 2. 66 ± 0. 05 to 2. 93 ± 0. 04 (*p* < 0. 05), and 3. 27 ± 0. 05 (*p* < 0. 001), respectively. Significant increases were also observed when comparing the 1000 mg/Kg group to both 250 mg/Kg group (2. 83 ± 0. 05 to 3. 27 ± 0. 05; *p* < 0. 001) and the 500 mg/Kg group (*p* < 0. 01). Similarly, postbiotic administration significantly increased OCLN scores in the 500 and 1000 mg/Kg groups relative to the control (Figure 13B), from 2. 41 ± 0. 07 to 2. 91 ± 0. 06 (*p* < 0. 01) and 3.06 ± 0. 07 (*p* < 0. 001), respectively. Comparisons also revealed significant increases from the 250 mg/Kg group (2.63 ± 0.05) to the 500 mg/Kg (*p* < 0.05), and 1000 mg/Kg groups (*p* < 0.01).

Regarding ZO1, postbiotic administration significantly increased scores in all groups compared to the control (Figure 13C), rising from 2 ± 0.1 to 2.54 ± 0.05 at 250 mg/Kg (*p* < 0.01), 2.68 ± 0. 05 at 500 mg/Kg (*p* < 0.01), and 2.93 ± 0.06 at 1000 mg/Kg (*p* < 0. 001). A significant difference was also observed between the 250 to the 1000 mg/Kg groups (*p* < 0.01). For AQP4, scores significantly increased in all except the 250 mg/Kg group, rising from 2.58 ± 0.07 to 2.89 ± 0.06 at 500 mg/Kg (*p* < 0.05), and 3.06 ± 0.06 at 1000 mg/Kg (*p* < 0.001). A significant difference was also noted between the 250 and 1000 mg/Kg groups (*p* < 0.01). With regard to AQP8, scores significantly increased across all groups compared to the control, rising from 1.2 ± 0.09 to 2.14 ± 0.06 at 250 mg/Kg (*p* < 0.05), to 2.68 ± 0.05 at 500 mg/Kg (*p* < 0.001), and to 2.83 ± 0.05 at 1000 mg/Kg (*p* < 0.001). Similar trends were observed when comparing the 250 mg/Kg group to both the 500 mg/Kg (*p* < 0.001) and 1000 mg/Kg groups (*p* < 0.01).

Significant positive correlations proportional to the dosage were detected for all biomarkers. The 250 mg/Kg dosage significantly increased OCLN, AQP4, and AQP8, the 500 mg/Kg dosage significantly increased the expression of all biomarkers compared to the control group (OR: 2.73–4.14). The 1000 mg/Kg dosage produced the most pronounced increases in protein expression (OR: 7.85–9.60). Comparison of immunohistochemical evaluation scores in intestinal tissues at different application times of postbiotics (days 7, 14, 21, and 28) is presented in Table S1 and Figure S4.

These results demonstrate a clear dose–response relationship. Additionally, the postbiotic application periods (days 7, 14, 21, 28) did not significant affect biomarker scores (Table 5).

## 4. Discussion

The efficacy of postbiotic products depends on the metabolic capacity of the microbial strain and the optimization of the production process. The high bacterial count of 8.48 log CFU/g recorded during the production indicates intense microbial metabolic activity in the fermentation medium, leading to the sufficient synthesis of bioactive compounds. Indeed, high cell densities have been shown to positively affect metabolite production and final product quality [27,28]. The 6.8% yield obtained in the present study is consistent with the generally reported range of 5–10% [18,29,30].

pH is a critical factor for the biological activity and stability of postbiotics. The measured acidic pH indicates successful organic acid production, consistent with the ability of *P. acidilactici* to ferment carbon sources. Lactic acid disrupts microbial cell membrane integrity by lowering pH, while acetic acid inhibits intracellular enzyme systems. The high levels of these two acids identified here suggest that the postbiotic possesses strong antimicrobial and protective potential. Furthermore, the detection of propionic, tartaric, and citric acids reveals a complex metabolic profile, similar to other postbiotic samples [22,31,32].

Phenolic compound analysis identified naringin (13.62 ng/µL), vanillin (8.85 ng/µL), and chlorogenic acid (5.17 ng/µL) as the primary compounds, significantly contributing to the antioxidant capacity. Naringin, a flavonoid known for free radical-scavenging ability and lipid-oxidation-inhibiting effect, likely drives the strong antioxidant effect shown in Table 1. Similar studies on *P. acidilactici* postbiotics have reported high total phenolic content associated with pronounced antioxidant activity [18,22,33,34].

GC-MS analysis identified 49 volatile compounds; acetic acid was the most abundant (43.68%), followed by benzaldehyde (11.60%), butanal, 3-methyl-, and pyrazine derivatives. These compounds define the postbiotics’ characteristic odor and contribute to its antimicrobial and antioxidant effects, consistent with existing literature [18,22,31,35].

While numerous in vivo studies on probiotics exist [36,37], research on postbiotics remains limited due to the novelty of the concept. As postbiotics lack live bacteria, they are increasingly being integrated into functional foods (e.g., postbiotic tea). Therefore, it is essential to determine their effects on the intestines of healthy individuals. This study evaluated the effects of *P. acidilactici-*derived postbiotics on intestinal homeostasis and mucosal barrier integrity by examining tight junction proteins (CLDN3, OCLN, and ZO1) and water transport proteins (AQP4 and AQP8).

In previous models, *Streptococcus thermophilus* and *Lactobacillus* (*L.*) *acidophilus* prevented the decrease in OCLN and ZO1 phosphorylation induced by *Escherichia* (*E.*) *coli* infection [38]. Similarly, *L. casei* modulated ZO1 expression to reinforce intercellular connections [39]. In our study, the dose-dependent increase in OCLN and CLDN3 expression indicates that postbiotics enhance the control of paracellular permeability. The parallel increase in ZO1 expression further optimizes intestinal physiology. Given that decreased OCLN is a marker for increased permeability and chronic inflammation, often seen in ulcerative colitis and Crohn’s disease [35,40], the dose-dependent increase observed here suggests that postbiotics may be effective in managing intestinal disorders.

Unlike studies where *L. plantarum* showed no effect on permeability in healthy subjects [36], our findings align more closely with Karczewski et al. (2010), who reported that *L. plantarum* WCFS1 increased OCLN and ZO1 expression in healthy intestines [41]. Interestingly, the lack of a significant “day” effect that the postbiotic’s impact is dose-dependent rather than cumulative process. This indicates a “receptor saturation” mechanism, where the immediate dosage determines the response rather than time-based accumulation.

AQP4 and AQP8 are essential for cellular fluid transport in the intestines [4]. Decreased aquaporin levels are frequently associated with diarrhea; for example, AQP2 and AQP3 were absent in the colons of individuals infected with enterohemorrhagic *E. coli* and enteropathogenic *E. coli* [42], and AQP4 expression drops significantly in colitis models [43]. Wang et al. (2007) also noted reduced AQP8 in irritable bowel syndrome patients with loose stools and diarrhea [44]. In this study, AQP4 and AQP8 expression increased with the postbiotics dosage. These elevated levels suggest that postbiotics enhance water absorption and trans-epithelial fluid balance, particularly in conditions where such balance is disrupted.

The chemical profile offers a mechanistic basis of these immunohistochemical findings. Bioactive compounds like naringin and vanillin protect the epithelial barrier by modulating oxidative stress and inflammatory signaling, which in turn regulates the expression of tight junction proteins [45]. Similarly, the regulation of AQP4 and AQP8 is closely linked to cellular integrity and stress levels [46]. In summary, the antioxidant and barrier-protective metabolites in the study may be related to the potential effects of bioactive compounds in the *P. acidilactici* postbiotic play a vital role in maintaining intestinal epithelial physiology and homeostasis.

## 5. Conclusions

In conclusion, this study investigated the effects of *P. acidilactici*-derived postbiotics on intestinal tissue at the cellular level. Immunohistochemical observations revealed that these postbiotics induced a dose-dependent increase in biomarkers responsible for both transepithelial and cellular transport. Based on these data, it is concluded that postbiotic use has direct positive effects on maintaining and strengthening intestinal barrier integrity while supporting overall intestinal homeostasis.

These findings are significant in reinforcing the clinical potential of postbiotics as a safe and stable alternative to probiotics. The results shed light on the development of supportive therapeutic strategies, particularly for chronic inflammatory bowel diseases such as Crohn’s disease and ulcerative colitis. Future research is recommended to elucidate the molecular signaling pathways underlying these positive effects and to further validate them using advanced preclinical models.

## Figures and Tables

**Table 1:** Some characterization parameters of the postbiotic derived from *P. acidilactici*.

Characterization Parameters	Results
Bacterial count	8.48 log CFU/mL
pH value	4.54 ± 0.11
Postbiotic yield (%)	6.8 ± 0.12 (6.8 mg/1 mL)
Total phenolic content	15,200 ± 4.30 GAE (µg/100 g dry matter)
DPPH radical scavenging activity (IC_50_)	78.6935 ± 6.32 µg/mL
ABTS radical scavenging activity (IC_50_)	6.274 ± 0.63 µg/mL

**Table 2:** Organic acids and their amounts contained in the postbiotic derived from *P. acidilactici* (Mean ± SD).

No	Organic Acid	Retention Time (min.)	Concentration (mg/mL)
1	Citric acid	9.24	8.69 ± 0.060
2	Tartaric acid	9.36	4.63 ± 0.030
3	Lactic acid	13.57	6.42 ± 0.020
4	Acetic acid	15.69	3.46 ± 0.030
5	Fumaric acid	17.26	0.002 ± 0.001
6	Propionic acid	19.26	0.170 ± 0.010

**Table 3:** Phenolic and flavonoid compounds and their amounts in the postbiotic derived from *P. acidilactici* (Mean ± SD).

No	Compound Name	Retention Time (min.)	Concentration (mg/L)
1	Chlorogenic acid	6.01	5.17 ± 0.03
2	Caffeic acid	9.29	2.76 ± 0.01
3	Vanillin	16.02	8.85 ± 0.04
4	p-Coumaric acid	17.40	1.35 ± 0.03
5	Hydroxycinnamic acid	23.28	1.54 ± 0.01
6	Naringin	27.41	13.62 ± 0.06
7	o-Coumaric acid	28.80	4.61 ± 0.02
8	Resveratrol	32.43	0.18 ± 0.03

**Table 4:** Volatile compound profile of postbiotic derived from *P. acidilactici* (% area ± SD).

Compound Group	Compound Name	Retention Time (min.)	Area (%)
Acids (9)	Acetic acid	16.748	43.68 ± 1.86
	Propanoic acid	19.569	0.12 ± 0.01
	Propanoic acid, 2-methyl-	20.494	0.42 ± 0.03
	Butanoic acid	22.265	1.99 ± 0.13
	Iso-valeric acid	23.474	0.44 ± 0.03
	Hexanoic acid	28.284	1.02 ± 0.22
	Octanoic acid	33.656	0.92 ± 0.06
	Nonanoic acid	36.152	0.11 ± 0.01
	n-Decanoic acid	38.545	0.38 ± 0.02
Alcohols (10)	2-Propanol	1.044	0.22 ± 0.01
	1-Propanol, 2-methyl-	4.986	0.18 ± 0.04
	1-Butanol	6.706	0.81 ± 0.06
	1-Butanol, 3-methyl-	8.868	4.25 ± 0.36
	1-Heptanol	17.168	0.11 ± 0.01
	1-Hexanol, 2-ethyl-	18.253	1.85 ± 0.28
	1-Octanol	20.341	0.2 ± 0.01
	1-Nonanol	23.334	0.22 ± 0.03
	Benzyl alcohol	28.973	0.18 ± 0.01
	Benzeneethanol	29.867	0.43 ± 0.06
Aldehydes (10)	Butanal, 3-methyl-	0.785	5.33 ± 0.46
	Hexanal	4.372	0.18 ± 0.01
	Heptanal	7.759	0.25 ± 0.01
	2-Butenal, 3-methyl-	8.144	0.17 ± 0.01
	Nonanal	14.854	0.5 ± 0.03
	Benzaldehyde	18.737	11.6 ± 0.72
	Nonenal	19.308	0.13 ± 0.01
	Benzeneacetaldehyde	22.303	2.96 ± 0.26
	(1-Bromocyclohexane)carboxaldehyde ethyl methyl acetal	25.018	0.2 ± 0.01
	Benzaldehyde, 4-propyl-	27.409	0.16 ± 0.01
Ketones (4)	2-Butanone	0.595	0.62 ± 0.06
	2-Butanone, 3-methyl-	1.769	0.25 ± 0.02
	2-Propanone, 1-hydroxy-	11.61	0.70 ± 0.02
	2-Acetylthiazole	22.532	0.53 ± 0.04
Esters (3)	Tetradecanoic acid, methyl ester	32.381	0.15 ± 0.01
	Hexadecanoic acid, methyl ester	37.233	0.34 ± 0.02
	9-Octadecenoic acid, methyl ester	42.112	0.15 ± 0.01
Nitrogen Compounds (9)	Pyrazine	8.638	0.91 ± 0.06
	Pyrazine, methyl-	10.518	4.67 ± 0.45
	Pyrazine, 2,5-dimethyl-	12.475	6.48 ± 0.39
	Pyrazine, 2,6-dimethyl-	12.691	3.16 ± 0.27
	Pyrazine, ethyl-	12.855	0.61 ± 0.03
	Pyrazine, 2-ethyl-6-methyl-	14.609	0.11 ± 0.01
	Pyrazine, trimethyl-	15.244	0.48 ± 0.02
	Pyrazine, 3-ethyl-2,5-dimethyl-	16.594	0.38 ± 0.04
	Pyrazine, 2,5-dimethyl-3-(3-methylbutyl)-	23.09	0.13 ± 0.01
Sulfur Compounds (2)	Disulfide, dimethyl	4.016	0.21 ± 0.01
	1-Propanol, 3-(methylthio)-	24.768	0.14 ± 0.01
Phenolic Compounds (1)	Phenol, 2,4-bis(1,1-dimethylethyl)-	39.387	0.49 ± 0.04
Siloxanes (1)	Cyclotrisiloxane, hexamethyl-	0.145	0.11 ± 0.01

**Table 5:** Ordinal regression results for immunohistochemical scores related to biomarkers according to tissue, day, and dose.

Predictor #	CLDN3		OCLN		ZO1		AQP4		AQP8	
	Estimate (SE)	OR (95%CI)	Estimate (SE)	OR (95%CI)	Estimate (SE)	OR (95%CI)	Estimate (SE)	OR (95%CI)	Estimate (SE)	OR (95%CI)
Jejunum	0.10 (0.31)	1.11 (0.61–2.02)	0.29 (0.30)	1.33 (0.75–2.38)	0.45 (0.30)	1.56 (0.88–2.80)	−0.05 (0.30)	0.95 (0.53–1.70)	−0.38 (0.30)	0.68 (0.38–1.22)
Ileum	1.07 (0.30) ***	2.92 (1.61–5.31)	0.28 (0.30)	1.32 (0.74–2.38)	−0.38 (0.29)	0.68 (0.38–1.21)	−0.89 (0.30) **	0.41 (0.23–0.74)	0.47 (0.30)	1.61 (0.90–2.87)
Cecum	0.48 (0.30)	1.62 (0.91–2.90)	−1.20 (0.31) ***	0.30 (0.16–0.55)	−0.79 (0.30) **	0.45 (0.25–0.81)	1.22 (0.31) ***	3.39 (1.87–6.19)	−1.79 (0.31) ***	0.17 (0.09–0.30)
Colon	1.48 (0.31) ***	4.39 (2.41–8.06)	−1.37 (0.31) ***	0.25 (0.14–0.46)	−1.08 (0.30) ***	0.34 (0.19–0.61)	0.65 (0.30) *	1.91 (1.07–3.44)	−1.78 (0.31) ***	0.17 (0.09–0.31)
Rectum	0.93 (0.30) **	2.52 (1.41–4.53)	−0.32 (0.30)	0.73 (0.40–1.32)	−1.02 (0.30) ***	0.36 (0.20–0.65)	0.56 (0.30)	1.74 (0.98–3.12)	−1.03 (0.30) ***	0.36 (0.20–0.64)
Day 14	−0.26 (0.24)	0.77 (0.48–1.25)	−0.01 (0.25)	0.99 (0.61–1.62)	−0.47 (0)	0.63 (0.39–1.01)	−0.19 (0.25)	0.83 (0.51–1.34)	−0.55 (0.24)	0.58 (0.36–0.93)
Day 21	−0.05 (0.24)	0.96 (0.60–1.53)	−0.05 (0.25)	0.95 (0.59–1.55)	−0.28 (0)	0.76 (0.47–1.22)	−0.13 (0.25)	0.88 (0.54–1.42)	−0.18 (0.24)	0.84 (0.53–1.34)
Day 28	−0.01 (0.24)	0.99 (0.62–1.61)	0.06 (0.25)	1.06 (0.65–1.71)	−0.17 (0)	0.84 (0.52–1.35)	0.10 (0.25)	1.11 (0.68–1.79)	−0.31 (0.24)	0.73 (0.45–1.18)
250 mg/kg	0.46 (0.28)	1.58 (0.92–2.73)	0.67 (0.28) *	1.96 (1.13–3.40)	0.43 (0.30)	1.53 (0.88–2.69)	0.96 (0.29) **	2.62 (1.48–4.68)	0.66 (0.28) *	1.94 (1.12–3.37)
500 mg/kg	1.27 (0.28) ***	3.58 (2.07–6.24)	1.30 (0.29) ***	3.69 (2.10–6.53)	1.00 (0.29) ***	2.73 (1.56–4.81)	1.42 (0.30) ***	4.13 (2.32–7.43)	1.42 (0.29) ***	4.14 (2.38–7.29)
1000 mg/kg	2.15 (0.30) ***	8.55 (4.82–15.35)	2.14 (0.30) ***	8.52 (4.79–15.35)	2.15 (0.30) ***	8.60 (4.80–15.65)	2.26 (0.31) ***	9.60 (5.32–17.62)	2.06 (0.29) ***	7.85 (4.44–14.03)
Model	χ^2^ = 109, df = 11, *p* < 0.001, R^2^_MacFadden_ = 0.088		χ^2^ = 113, df = 11, *p* < 0.001, R^2^_MacFadden_ = 0.096		χ^2^ = 111, df = 11, *p* < 0.001, R^2^_MacFadden_ = 0.092		χ^2^ = 114, df = 11, *p* < 0.001, R^2^_MacFadden_ = 0.096		χ^2^ = 144, df = 11, *p* < 0.001, R^2^_MacFadden_ = 0.114	

#: Reference categories were determined as duodenum for tissue, day 7 for days, and 0 mg/kg for dose; SE: Standard Error of Estimate; OR: Odds Ratio; CI: Confidence Interval; χ^2^: Chi-Square; R^2^: Coefficient of Determination; * *p* < 0.05, ** *p* < 0.01, *** *p* < 0.001.

## Data Availability

The original contributions presented in this study are included in the article. Further inquiries can be directed to the corresponding authors.

## Supplementary Materials

The following supporting information can be downloaded at: https://www.mdpi.com/article/10.3390/foods15071267/s1, Figure S1: Chromatogram of organic acids contained in postbiotic derived from *Pediococcus acidilactici*; Figure S2: Chromatogram of phenolic and flavonoid compounds contained in the postbiotic derived from *Pediococcus acidilactici*; Figure S3: Chromatogram of volatile compounds contained in the postbiotic derived from *Pediococcus acidilactici*; Figure S4: Immunohistochemical evaluation scores of intestinal biomarkers across different application periods of postbiotics; Table S1: Comparison of immunohistochemical evaluation scores across different application periods (days 7, 14, 21, and 28) in intestinal tissues (Kruskal–Wallis test).

## Abbreviations

The following abbreviations are used in this manuscript:

OCLN	Occludin
CLDN	Claudin
JAM	Junctional adhesion molecule
AQP4	Aquaporin 4
AQP8	Aquaporin 8
RP-HPLC	Reverse-phase high-performance liquid chromatography
GC–MS	Chromatography-mass spectrometry
PBS	Phosphate-buffered saline
ZO1	Zonula occludens-1
CLDN3	Claudin-3
DAB	3-3′ Diaminobenzidine
