# Supplementary material for: Investigation of the Effects of Postbiotics Obtained from Pediococcus acidilactici on Specific Biomarker Expressions in Intestinal Tissue
Source: Foods. 2026 Apr 7;15(7):1267. doi: 10.3390/foods15071267 (PMC13073207; doi:10.3390/foods15071267)
Supplement: Supplementary file 1 [file foods-15-01267-s001.zip › foods-4196176-supplementary.pdf]

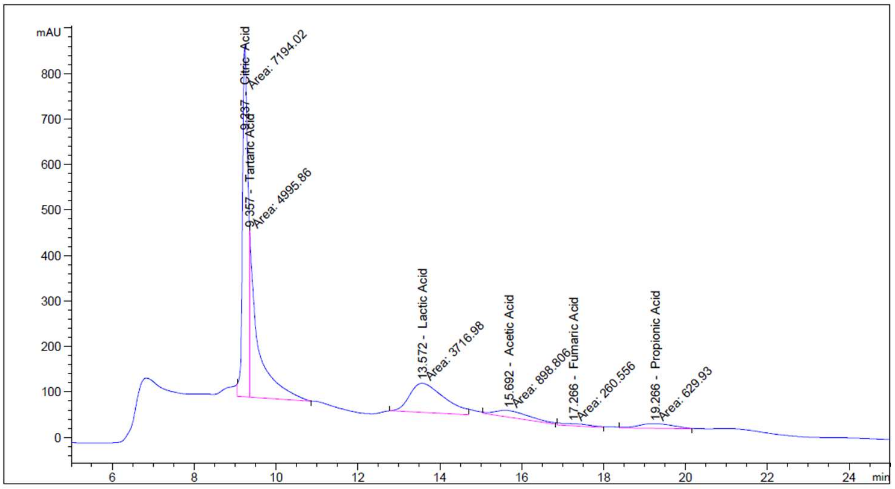

Figure S1. Chromatogram of organic acids contained in postbiotic derived from *Pediococcus acidilactici*.

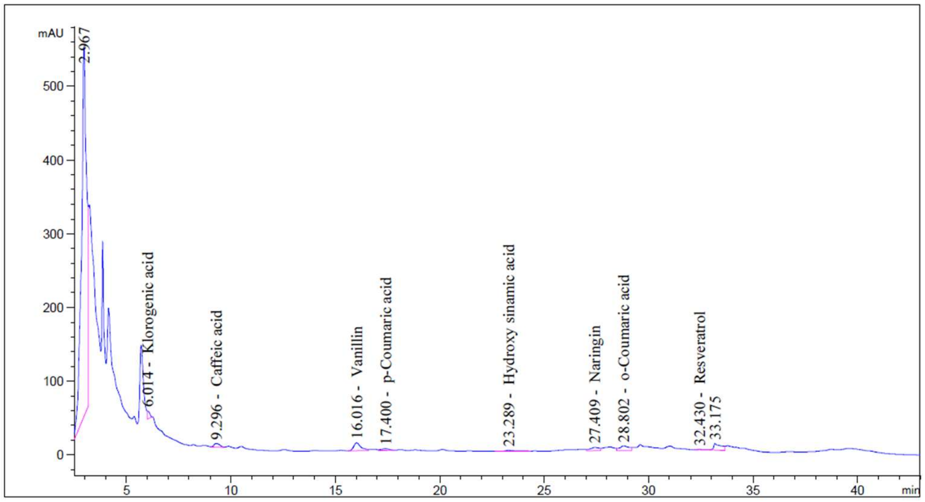

Figure S2. Chromatogram of phenolic and flavonoid compounds contained in the postbiotic derived from *Pediococcus acidilactici.*

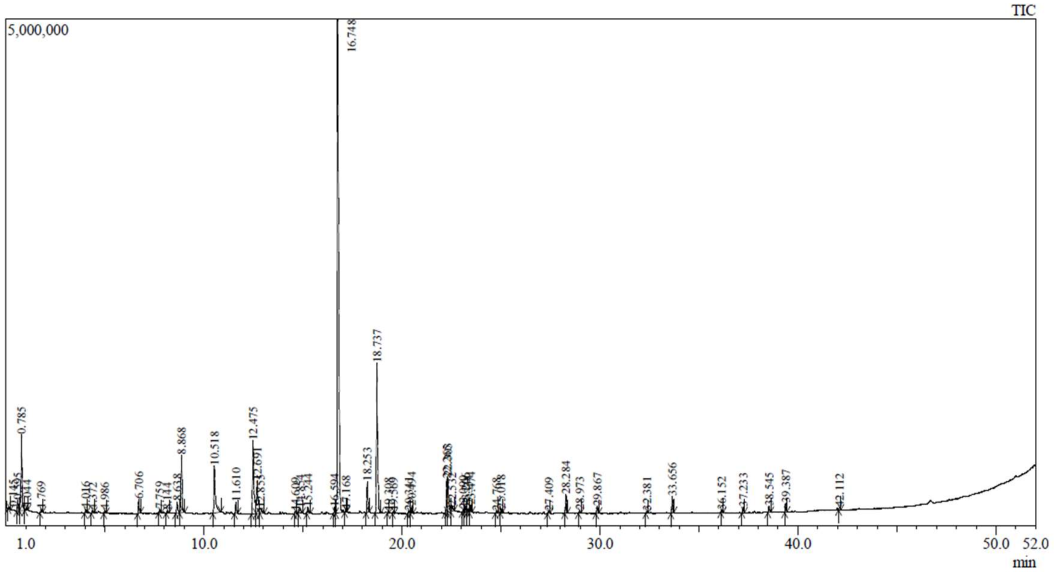

Figure S3. Chromatogram of volatile compounds contained in the postbiotic derived from *Pediococcus acidilactici.*

Table S1. Comparison of immunohistochemical evaluation scores across different application periods (days 7, 14, 21, and 28) in intestinal tissues (Kruskal–Wallis test).

Tissues	Proteins	7 day	14 day	21 day	28 day	P value
Duedonum	Cl	2.74±0.19	2.60±0.17	2.60±0.18	2.62±0.12	0.973
	OC	3.05±0.18	2.95±0.18	2.98±0.17	2.83±0.14	0.881
	ZN	3.24±0.19	2.95±0.19	3.12±0.18	2.90±0.15	0.716
	AQ4	2.79±0.21	2.64±0.12	2.52±0.17	2.69±0.13	0.840
	AQ8	3.12±0.20	2.83±0.15	3.02±0.18	2.98±0.17	0.792
Jejenum	Cl	2.74±0.22	2.50±0.23	2.71±0.19	2.64±0.22	0.810
	OC	3.02±0.14	3.00±0.13	3.00±0.14	3.10±0.16	0.970
	ZN	3.21±0.18	3.14±0.18	3.14±0.19	3.33±0.19	0.832
	AQ4	2.48±0.21	2.31±0.18	2.38±0.14	2.50±0.19	0.928
	AQ8	3.33±0.18	3.07±0.18	3.29±0.17	3.33±0.20	0.663
Ileum	Cl	3.12±0.19	2.98±0.19	3.00±0.15	3.02±0.21	0.957
	OC	3.21±0.17	2.98±0.16	3.00±0.17	3.07±0.17	0.759
	ZN	3.00±0.19	2.86±0.18	2.83±0.15	2.93±0.18	0.912
	AQ4	2.81±0.17	2.60±0.16	2.67±0.16	2.69±0.18	0.837
	AQ8	2.95±0.19	2.86±0.18	2.86±0.14	2.90±0.21	0.979
Cecum	Cl	2.90±0.15	2.71±0.15	2.74±0.12	2.86±0.16	0.765
	OC	2.26±0.17	2.52±0.15	2.36±0.17	2.64±0.14	0.331
	ZN	2.76±0.17	2.62±0.13	2.74±0.14	2.69±0.18	0.905
	AQ4	2.93±0.14	2.86±0.16	2.90±0.16	2.90±0.17	0.990
	AQ8	2.79±0.15	2.57±0.19	2.60±0.12	2.67±0.21	0.768
Colon	Cl	3.05±0.16	3.10±0.19	3.29±0.18	3.31±0.19	0.656
	OC	2.36±0.17	2.38±0.17	2.40±0.19	2.60±0.13	0.647
	ZN	2.60±0.15	2.45±0.15	2.62±0.15	2.71±0.17	0.699
	AQ4	2.98±0.15	3.14±0.17	3.17±0.18	3.29±0.19	0.638
	AQ8	2.29±0.14	2.26±0.18	2.38±0.14	2.45±0.16	0.826
Rectum	Cl	2.93±0.13	2.95±0.14	2.95±0.14	3.02±0.14	0.968
	OC	2.83±0.18	2.74±0.16	2.83±0.14	2.83±0.19	0.970
	ZN	2.67±0.16	2.57±0.14	2.57±0.19	2.67±0.18	0.956
	AQ4	2.93±0.14	2.90±0.17	2.90±0.18	2.93±0.17	0.999
	AQ8	2.36±0.16	2.38±0.18	2.36±0.18	2.33±0.19	0.998

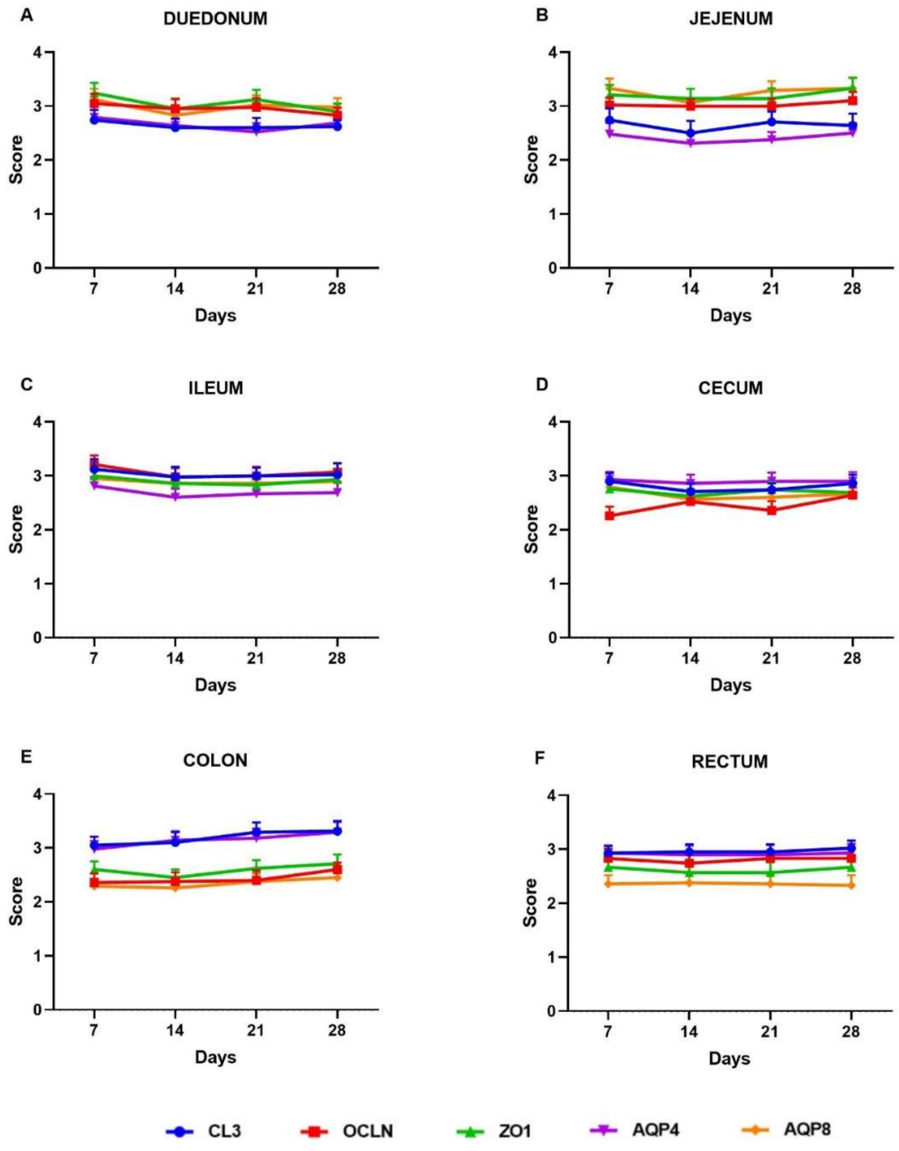

Figure S4. Immunohistochemical evaluation scores of intestinal biomarkers across different application periods of postbiotics. Panels represent intestinal segments: (A) Duodenum, (B) Jejunum, (C) Ileum, (D) Cecum, (E) Colon, and (F) Rectum. The x-axis indicates the application period (days 7, 14, 21, 28), while the y-axis represents immunohistochemical scores. Colored lines represent different proteins (CL3, OCLN, ZO1, AQP4, and AQP8). No statistically significant differences were detected among application periods for any biomarker (Kruskal–Wallis test). These findings indicate that the duration of postbiotic administration did not significantly influence biomarker expression levels.
